# Transient reduction of DNA methylation at the onset of meiosis in male mice

**DOI:** 10.1186/s13072-018-0186-0

**Published:** 2018-04-04

**Authors:** Valeriya Gaysinskaya, Brendan F. Miller, Chiara De Luca, Godfried W. van der Heijden, Kasper D. Hansen, Alex Bortvin

**Affiliations:** 1 Department of Embryology, Carnegie Institution for Science, Baltimore, MD USA; 2 Department of Biology, Johns Hopkins University, Baltimore, MD USA; 3 Translational and Functional Genomics Branch, National Human Genome Research Institute, National Institutes of Health, Bethesda, MD USA; 4 Department of Obstetrics and Gynaecology, Erasmus MC, University Medical Center, PO BOX 2040, 3000 CA Rotterdam, The Netherlands; 5 Department of Biostatistics, Johns Hopkins University, Baltimore, MD USA; 6 Center for Computational Biology, Johns Hopkins University, Baltimore, MD USA; 7 McKusick-Nathans Institute of Genetic Medicine, Johns Hopkins University, Baltimore, MD USA

**Keywords:** DNA methylation, Hemimethylation, Spermatogenesis, LINE-1, Retrotransposon, Mouse, Meiosis, Meiotic prophase

## Abstract

**Background:**

Meiosis is a specialized germ cell cycle that generates haploid gametes. In the initial stage of meiosis, meiotic prophase I (MPI), homologous chromosomes pair and recombine. Extensive changes in chromatin in MPI raise an important question concerning the contribution of epigenetic mechanisms such as DNA methylation to meiosis. Interestingly, previous studies concluded that in male mice, genome-wide DNA methylation patters are set in place prior to meiosis and remain constant subsequently. However, no prior studies examined DNA methylation during MPI in a systematic manner necessitating its further investigation.

**Results:**

In this study, we used genome-wide bisulfite sequencing to determine DNA methylation of adult mouse spermatocytes at all MPI substages, spermatogonia and haploid sperm. This analysis uncovered transient reduction of DNA methylation (TRDM) of spermatocyte genomes. The genome-wide scope of TRDM, its onset in the meiotic S phase and presence of hemimethylated DNA in MPI are all consistent with a DNA replication-dependent DNA demethylation. Following DNA replication, spermatocytes regain DNA methylation gradually but unevenly, suggesting that key MPI events occur in the context of hemimethylated genome. TRDM also uncovers the prior deficit of DNA methylation of LINE-1 retrotransposons in spermatogonia resulting in their full demethylation during TRDM and likely contributing to the observed mRNA and protein expression of some LINE-1 elements in early MPI.

**Conclusions:**

Our results suggest that contrary to the prevailing view, chromosomes exhibit dynamic changes in DNA methylation in MPI. We propose that TRDM facilitates meiotic prophase processes and gamete quality control.

**Electronic supplementary material:**

The online version of this article (10.1186/s13072-018-0186-0) contains supplementary material, which is available to authorized users.

## Background

Meiosis is a specialized cell division program that produces haploid gametes. To achieve haploidy, a diploid germ cell replicates its DNA once and divides twice. Following the final round of DNA replication (meiotic S phase), chromosomes pair and recombine in meiotic prophase I (MPI) [1]. Meiosis is a highly protracted cell cycle due to meiotic S phase being much longer than mitotic S phase in the same organism [2, 3] and MPI itself lasting about 2 weeks [4], during which time the chromosomes undergo dramatic changes in organization. Based on these changes, MPI is subdivided into leptotene (L), zygotene (Z), pachytene (P) and diplotene (D) substages, which are immediately preceded by meiotic S phase also known as the preleptotene (PL) stage (Additional file 1: Fig. S1). These descriptive names of MPI substages serve as common reference points in the studies of meiosis across species since they associate with specific molecular processes such as double-stranded break (DSB) formation in L, the onset of homolog synapsis in Z, completion of synapsis and meiotic recombination in P and the onset of homolog decondensation and desynapsis in D. Upon the completion of MPI, homologous chromosomes segregate in the first meiotic division (Meiosis I), while sister chromatids separate in Meiosis II.

Extensive changes in meiotic chromosome configurations in MPI raise an important question concerning the contribution of epigenetic mechanisms to meiosis. Indeed, prior studies have implicated histone modifications in chromosome condensation, synapsis and meiotic recombination in plants, fungi and animals [5–9]. Disruption of DNA methylation also interferes with MPI processes in a wide range of species capable of this modification including mammals [10–15]. Here, we focused on genome-wide DNA methylation of male meiotic germ cells of mice. Studies over the past decade revealed important roles of DNA methylation, repressive histone modifications and small Piwi-interacting RNAs (piRNAs) in LINE-1 (L1) control in male germ cells [13, 16–18]. Intriguingly, despite these defensive mechanisms, early meiotic male germ cells exhibit L1 ORF1p expression albeit at levels significantly lower than those observed in mutants deficient in transposon defense [18–22]. To explain this observation, we have previously posited a transient change in DNA methylation at the onset of meiosis [23]. Critically, unlike the detailed knowledge of genome-wide DNA methylation in mouse postnatal spermatogonia [24, 25] and the evidence that bulk DNA methylation precedes meiosis [26], the precise dynamics of DNA methylation during MPI remain unknown. To a large extent, this gap in understanding of epigenetic makeup of meiotic chromosomes was due to inaccessibility of cell populations from all MPI substages. To overcome this limitation, we first have optimized the method for the purification of adult mouse male germ cells from all substages of MPI [27–29]. In this study, using this method, we obtained high-quality germ cell samples that allowed us to discover genome-wide transient reduction of DNA methylation (TRDM) during MPI, a previously unrecognized epigenetic feature of meiotic chromosomes in male mice.

## Results

### Genome-wide DNA methylation levels in meiotic prophase I

To characterize the dynamics of DNA methylation across MPI, we used an optimized flow cytometry cell sorting method to obtain two biological replicates of spermatogonial (Spg), PL, L, Z, P, D spermatocytes and epididymal spermatozoa (Spz) [27–29] (Additional file 2: Fig. S2). The purity of MPI cell fractions was verified by staining for meiosis-specific (SYCP3, γH2AX) and spermatogonia-enriched (DMRT1, DMRT6) markers as described previously [28, 29]. Critically, all germ cell fractions were devoid of somatic cells (Methods) and gene expression profiling of a wide panel of soma-enriched and germ cell-specific genes by RNA-seq confirmed the purity and stage specificity of our samples (Additional file 3: Fig. S3). Using these samples, we performed whole-genome bisulfite DNA sequencing (WGBS) for genome-wide analysis of DNA methylation at single CpG resolution (Additional file 4: Table S1). Over 90% of reads aligned to the mouse genome and exhibited high efficiency of bisulfite conversion (Additional file 4: Table S1 and Additional file 5: Table S2). Each biological replicate accounted for 87–94% of genomic CpGs with 3 to 6 × average CpG coverage per individual sample after read de-duplication and processing (Additional file 6: Table S3). Pairs of biological replicates exhibited high inter-individual Pearson correlation indicating excellent reproducibility of our data (Additional file 7: Table S4). Since cytosine methylation levels at non-CpG sites were negligible (0.3–0.4%), we excluded them from later analyses.

**Fig. 1:**
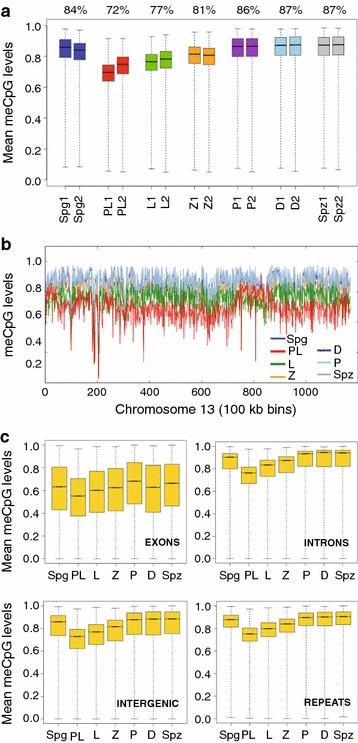
Global DNA methylation dynamics in MPI. **a** Genome-wide DNA methylation was summarized as means of non-overlapping bins of 500 CpGs for individual biological replicates. Box-and-whisker plot shows the maximum, upper quartile, median, lower quartile and minimum of data. Median percent DNA methylation for both replicates is specified above the boxplot. **b** Chromosome-wide DNA methylation levels were plotted across chromosome length (chromosome 13, replicate 1 is shown). DNA methylation was averaged using sliding non-overlapping bins of 100 kbp. **c** Box-and-whisker plot of DNA methylation levels across various genomic features. The average DNA methylation levels were aggregated as consecutive, non-overlapping averages of 100 CpGs. Averages were combined for biological replicates

First, we examined genome-wide DNA methylation levels across MPI. Consistent with the notion of high DNA methylation levels in spermatogenesis, we observed high median (> 84%) levels of CpG methylation in Spg, P, D and Spz genomes (Fig. 1a, Additional file 8: Table S5). Interestingly, we uncovered an extended window of reduced global DNA methylation during early MPI demarcated by a pronounced drop (~ 12–13 percentage points) in DNA methylation levels in PL followed by a progressive gain of DNA methylation in L and Z, returning to premeiotic levels by P (Fig. 1a, Additional file 8: Table S5). Overall, a period of reduced global DNA methylation lasts from PL to P (~ 70 h), and we will refer to it as transient reduction of DNA methylation at the onset of meiosis (TRDM).

To examine the chromosome-wide distribution of DNA methylation in individual MPI substages, we summarized DNA methylation levels over a distance of 100-kb-wide non-overlapping windows spanning the length of each chromosome. We found that global hypomethylation in PL is chromosome-wide (Fig. 1b). This was true for all autosomes examined in both biological replicates (Additional file 9: Fig. S4, Additional file 10: Fig. S5). Interestingly, although the X chromosome also exhibits TRDM, it tended to be less methylated in all MPI substages (Additional file 9: Fig. S4, Additional file 10: Fig. S5, Additional file 11: Fig. S6). X chromosome DNA methylation levels in Spg-to-PL and PL-to-L transitions are distinctly less correlated than in the autosomes, further suggesting differences in the dynamics of its demethylation and remethylation (Additional file 11: Fig. S6). Nonetheless, these results showed that TRDM holds true for all chromosomes and that remethylation in MPI appears as a gradual chromosome-wide process.

To determine whether DNA hypomethylation in PL is specific to a particular genomic feature, we examined DNA methylation dynamics of exons, introns, intergenic and repetitive regions, as well as functionally specialized sequences such as promoters and CpG islands (CGIs) (Fig. 1c, Additional file 12: Fig. S7A, Additional file 13: Table S6). This analysis showed that all genomic features were highly methylated in Spg and then demethylated in PL (most prominently at introns, repeats and intergenic regions), except for CGIs whose methylation levels are already very low. Likewise, the analysis revealed comparable DNA methylation dynamics of repetitive DNA with major classes of TEs, namely the LINEs, SINEs, LTRs and DNA transposons (Additional file 12: Fig. S7B). Finally, we asked whether differentially methylated regions (DMRs) of imprinted genes also become hypomethylated in PL. The analysis of a subset of imprinted DMRs [30] showed that DNA methylation levels of paternal imprinted DMRs follow the same dynamic observed for other genomic features while maternal DMRs remained unmethylated as expected (Additional file 12: Fig. 7C). Cumulatively, these results show that TRDM is indeed a genome-wide event that encompasses all chromosomes and all genomic features.

### Dynamics of DNA methylation in the course of MPI

**Fig. 2:**
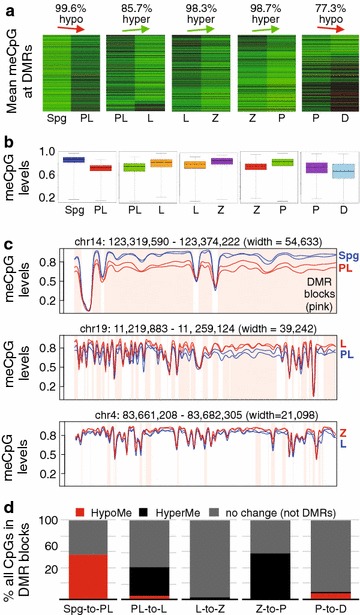
DMR dynamics in MPI. **a** Heatmap of DNA methylation profiles of all DMRs between consecutive MPI substages. Each row shows DNA methylation level of a DMR in a pairwise comparison. DNA methylation level is scaled according to red (low)-to-green (high) color scale. The percentage of DMRs exhibiting the main direction of DNA methylation change is indicated above the plots. **b** Boxplot showing DNA methylation value distribution at DMRs in MPI between two consecutive stages. **c** Smoothed DNA methylation at DMRs for Spg and PL (top), PL and L (middle), and P and D (bottom). **d** The proportion of CpGs accounted for by hypomethylated and hypermethylated DMRs

To better understand the DNA methylation dynamics in MPI, we identified regions that exhibited significant differences in methylation levels between any two consecutive MPI substages in a statistically principled, coverage-conscious and biological replicate-aware manner [31]. This analysis revealed thousands of DMRs supporting the results of our genome- and chromosome-wide analyses (Fig. 2, Additional file 14: Table S7 and Additional file 15: Table S8A). Formation of large hypomethylated DMRs (with a median size of ~ 35 kb, the median number of CpGs around 257 and implicating over half of the mouse genome) marked the Spg-to-PL transition (Fig. 2a, b). As a result of gradual remethylation of hypomethylated Spg-to-PL DMRs in L and Z, their mean methylation difference and sizes also progressively decreased (Fig. 2b, c). Thus, while Spg-to-PL DMRs included ~ 56% of all evaluated CpGs, PL-to-L and L-to-Z DMRs included ~ 41 and ~ 3% of all CpGs, respectively (Additional file 15: Table 8A). By intersecting genomic coordinates of DMRs between MPI substages, we found that Spg-to-PL DMRs accounted for up to 75% of all PL-to-L and 63% of L-to-Z DMRs (Methods). Therefore, a sharp demethylation in PL is followed by gradual remethylation of the same CpGs in L and Z germ cells.

Although global levels of DNA methylation in Z were higher relative to the preceding substages, Z is still hypomethylated relative to P (Fig. 2a). Accordingly, our DMR analysis showed that during the Z-to-P transition there is an increase in methylation at ~ 57% of analyzed CpGs from 81% in Z to 88% P (Additional file 15: Table S8A, Fig. 2b). Therefore, while the bulk of remethylation occurs by Z, remethylation that reaches premeiotic or almost Spz-like levels occurs between Z and P. Indeed, the original Spg-to-PL DMRs explain most (~ 75%) of all DMRs observed between Z and P (Methods). We find that gradual remethylation concerns all genomic features examined (exons, introns coding sequences and repeats) (Additional file 15: Table S8B). In Z, up to 60% of these features are still hypomethylated compared to P, although mean DNA methylation difference is relatively small (Fig. 2d, Additional file 15: Table S8A). In P, less than two percent of these features are found in hypomethylated DMRs relative to D, due to remethylation.

Interestingly, while P and D share very similar DNA methylation profiles overall (Fig. 1a), we observed the emergence of hypomethylated P-to-D DMRs that involve 8% of all examined CpGs in common and are of a relatively small mean genomic size (~ 9 kb, as compared to ~ 35 kb in PL) (Fig. 2a, b, d, Additional file 15: Table S8A). Considering that Spg-to-PL DMRs account for only 50% of all P-to-D DMRs (Additional file 15: Table S8A), it is likely that the hypomethylation observed in late MPI is unrelated to TRDM.

### Evidence of DNA replication-dependent DNA demethylation in TRDM

**Fig. 3:**
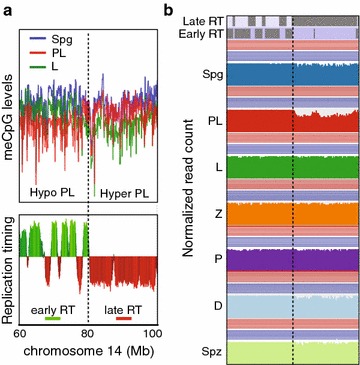
DNA methylation pattern in PL overlaps with replication timing. **a** The plot of CpG DNA methylation averaged using sliding non-overlapping 100-kbp windows in Spg, PL and L across a region of chromosome 14 (top) and replication timing (RT) data for the same region of chromosome 14 from mouse B cell lymphoma CH12 cells (bottom). **b** Normalized genome sequencing coverage after WGBS summarized as averages of sliding non-overlapping 5-kbp windows

The discovery of TRDM raised the question of its mechanistic origin. By visually inspecting patterns of chromosome-wide DNA methylation levels, we observed a possible clue to the cause of hypomethylation in PL. Focusing on PL DNA methylation trace along a chromosome, one can observe regions of relative DNA hypomethylation interrupted by a few prominent regions of relative hypermethylation (Fig. 3a, Additional file 9: Fig. S4, Additional file 10: Fig. S5). In fact, every chromosome in both PL biological replicates possessed such prominent subchromosomal domains (Additional file 9: Fig. S4, Additional file 10: Fig. S5, Additional file 16: Fig. S8, Additional file 17: Fig. S9A). Furthermore, the subchromosomal domains of higher relative DNA methylation levels in PL show lower DNA methylation levels in L, resulting in an apparent switch in DNA methylation traces in these MPI substages when compared to the rest of the chromosome (Fig. 3a top panel, Additional file 16: Fig. S8A, Additional file 17: Fig. S9A).

Given the above dynamics of DNA methylation in the PL-to-L transition, we considered a role for DNA replication and replication timing domains in this phenomenon. Replication domains are large-scale genomic territories that replicate at particular times during S phase [32, 33]. Global early or late replication timing profiles appear relatively preserved between different cell lines and cell types tested, although there are tissue-specific differences [33, 34]. Remarkably, an overlay of the chromosome-wide DNA methylation pattern from our data with replication timing domains of a mouse B cell lymphoma CH12 cell line [35] revealed a strong overlap between the two (Fig. 3a). Specifically, in PL, we observe an overlap between large hypermethylated regions and late-replicating domains. Correspondingly, an overlap is observed in PL between large-scale hypomethylated regions and early-replicating domains. Interestingly, a switch between DNA hypo- and hypermethylation in PL is marked by an opposite switch in DNA methylation pattern in L (Fig. 3a). This switch in DNA methylation pattern in PL-to-L transition matched the transition from early to late replication timing domains (Fig. 3a). The overlap between DNA methylation pattern and replication timing pattern in PL was true of both biological replicates (e.g., Fig. 3a, Additional file 16: Fig. S8, Additional file 17: Fig. S9). To test the strength of the association of a switch in DNA methylation levels with late replication genome-wide, we determined their Pearson correlation coefficient in the course of MPI. This analysis showed an abrupt switch in the directionality of correlation from PL to L, supporting that late-replicating domains switch from high to low DNA methylation levels between the two MPI stages (Additional file 18: Fig. S10A).

To further explore a role of DNA replication in hypomethylation of the PL genome, we evaluated the uniformity of genome sequencing coverage in our WGBS data (Fig. 3b). Previously, DNA sequence coverage was used to estimate replication timing and to evaluate underreplication in *Drosophila* polytene chromosomes [36, 37]. We summarized read frequency over a distance of 5-kb non-overlapping windows spanning the length of the chromosome and corrected for the difference in total read count between the samples. Remarkably, we observe consistently lower sequencing coverage in the hypermethylated regions/late replication timing domains in PL, disappearing in L (Fig. 3b, Additional file 16: Fig. S8, Additional file 17: Fig. S9). The lower sequencing coverage in PL is consistent with DNA replication during this time, while recovery of sequence coverage in L agrees with the lack of replication in L, as no replication occurs then and during the rest of meiosis. Specifically, lower sequencing coverage of late replication timing domains in PL indicates that these regions have not yet completed replication (with lower sequencing coverage reflecting lower DNA content), while early replication timing domains have already replicated (hence exhibit higher sequencing coverage associated with higher DNA content). To confirm that PL spermatocytes used in our studies are replicative, we performed FACS enrichment of PL cells from mice injected with EdU 2 h prior to cell sorting. Subsequent EdU detection showed that > 70% of FACS-enriched PL cells were replicative, with the majority of EdU patterns corresponding to middle and late S phase (Additional file 19: Fig. S10B) [38].

**Fig. 4:**
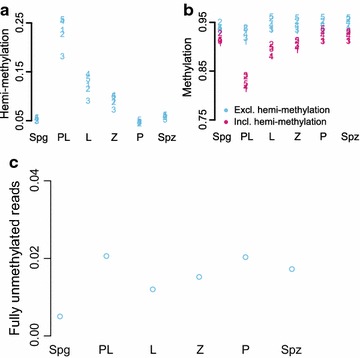
Hemimethylation of L1. Hemimethylated, methylated and unmethylated levels were quantified at 5 CpGs in L1. **a** The proportion of reads supporting hemimethylation at each of the 5 CpGs in six different cell states. **b** The amount of methylation at the 5 CpGs quantified including hemimethylation (pink) and excluding hemimethylation (blue). **c** Levels of fully unmethylated L1 elements in MPI based on reads where all 5 CpGs are unmethylated on both strands

The above results suggested that DNA replication in PL dilutes DNA methylation levels by creating hemimethylated DNA. To test this possibility directly, we analyzed methylation of complementary DNA strands using hairpin-bisulfite analysis [39] combined with next-generation sequencing (Methods). Here we focused on the 5`-end sequence of full-length L1 elements in L1MdTf_I and L1Md_Tf_II families [40]. The mouse genome has ~ 3000 of such elements, thus permitting simultaneous measurement of DNA hemimethylation throughout the genome. After PCR amplification of hairpin-bisulfite products using L1MdTf-specific primers (Additional file 19: Fig. S11) followed by Illumina paired-end sequencing of these L1MdTf amplicons, we obtained a range of 77–172 thousand reads per each analyzed MPI stage (Additional file 20: Table S9). We reliably determined methylation states of 5 L1 CpG dyads (Additional file 19: Fig. Fig. S11) before, during and after MPI. This analysis revealed a robust increase in L1 CpG hemimethylation in PL that was followed by gradual remethylation in MPI (Fig. 4a, b). Notably, L1 hemimethylation levels and dynamics in MPI paralleled those of LINE elements in our genome-wide DNA methylation studies (compare Fig. 4a, red values, with Additional file 12: Fig. S7B). Importantly, by excluding hemimethylated L1 DNA from this analysis we effectively almost erased TRDM of L1 (Fig. 4b, blue values). Together with the above evidence, these results strongly support the primary mechanism of TRDM by DNA replication-driven passive DNA demethylation. Finally, this analysis also revealed the emergence of L1s bearing no methylation on analyzed CpG (Fig. 4c), suggesting that TRDM reveals the existence of L1 elements incompletely methylated in Spgs, thus providing an opportunity for their expression at the onset of MPI.

### Transposon expression during TRDM

**Fig. 5:**
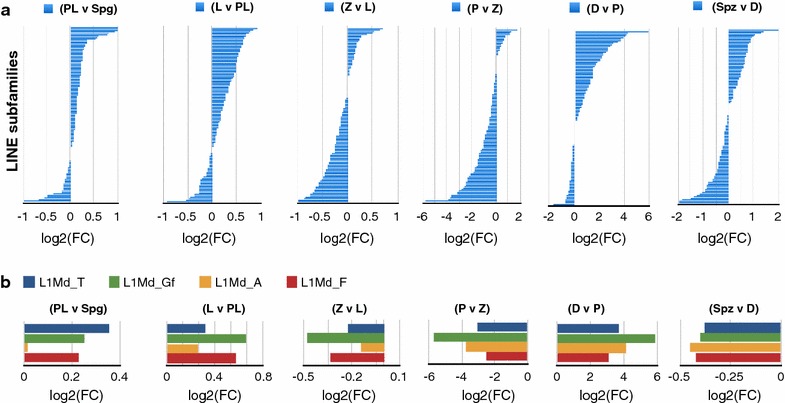
Dynamics of LINE transcript abundance in MPI.** a**, **b** A pairwise differential expression analysis is represented as log2 fold change in CPM between consecutive MPI stages and also sperm (Spz) relative to diplotene stage. Each horizontal barplot shows log2(FC) on the *x*-axis for **a** different LINE subfamily on the *y*-axis or **b** LINE-1 subfamilies that contain evolutionarily young and potentially active members

To test whether TRDM of potentially active L1s in PL contributes to their expression in MPI, we performed RNA-seq of FACS-enriched individual MPI cell populations (Methods). To analyze RNA abundance of TEs, we used RepEnrich strategy to account for most TE-derived RNA by way of counting both uniquely mapped and multi-mapped reads in our RNA-seq data [41]. Using this strategy, we found that transcript abundance for repeat elements as a whole shows an overall decrease from Spg onwards, with lowest levels in Spz (Additional file 21: Fig. S12). Intriguingly, we find that Spg-to-PL and PL-to-L transitions are accompanied by transcriptional upregulation of many classes of LINE elements (Fig. 5a). This upregulation includes all classes of potentially active L1 elements, whose expression begins to decrease in Z and is essentially extinguished by P (Fig. 5b**)**. Interestingly, a P-to-D transition involves a strong upregulation of LINEs including potentially active LINE members, a phenomenon apparently independent of TRDM (Fig. 5b).

To determine how these two bursts of L1 transcription relate to L1 protein expression, we performed immunofluorescence analysis using antibodies to the L1-encoded ORF1 protein, an acrosome-specific marker sp56 and double-strand break marker γH2AX [20, 42, 43]. This analysis established that L1ORF1p expression in MPI begins in L, persists until mid-P and extinguishes in late P (Additional file 22: Fig. S13**)**. These results suggest that the initial, smaller wave of L1 mRNA in the early MPI is productive, while the second burst of L1 transcription at P-to-D transition does not lead to a corresponding increase in L1ORF1p levels. These L1 mRNA and protein expression dynamics fit well with the relatively low activity of the piRNA pathway in early MPI and its robust transcriptional activation in P [18, 44].

### Expression analysis of DNA methylation machinery supports passive DNA methylation in TRDM

**Fig. 6:**
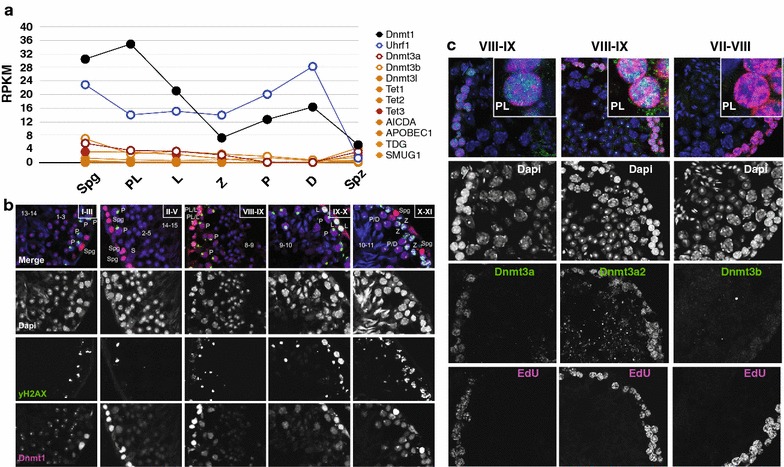
Examination of transcript and protein abundance of genes associated with passive or active DNA demethylation or remethylation. **a** RNA-seq transcript abundance of select genes in individual FACS-enriched MPI germ cells, expressed as RPKM. **b** Immunofluorescence co-staining of testicular cross sections for Dnmt1 (pink) and yH2AX (green), and counterstaining with DAPI (blue). The Roman numerals denote spermatogenic stages; the numbers correspond to steps of haploid sperm development during spermatogenesis. Spermatogonia (Spg), preleptonema (PL), leptonema (L) and zygonema (Z), pachynema (P), diplonema (D), Sertoli cells (S). **c** Immunofluorescence staining of testicular cross sections (cryosections) for EdU (pink) and Dnmt3a, Dnmt3a2 or Dnmt3b (green), and counterstaining with DAPI (blue)

To better understand the mechanism of TRDM, we examined MPI expression of genes associated with passive and active DNA demethylation. In cultured mammalian cells, maintenance CpG methylation in a newly synthesized DNA strand occurs within minutes after DNA replication [45, 46]. This process requires targeting of Dnmt1 to chromatin containing hemimethylated DNA by Uhrf1 protein [47, 48]. Our expression data indeed support dynamic but appreciable *Dnmt1* mRNA and protein expression in MPI (Fig. 6a, b). Interestingly, *Uhrf1* mRNA expression levels drop twofold in PL, L and Z compared to Spgs (Fig. 6a), thus providing a potential explanation to diffuse nucleoplasmic rather than replication foci-centered localization of Dnmt1 protein in PL (Fig. 6b and [49]). These observations agree with passive DNA demethylation in early MPI and are further supported by reduced *Dnmt1* expression in L and Z (Fig. 6a).

At the same time, genes for proteins implicated in active DNA demethylation exhibit low level of expression across MPI arguing against the leading role of this mechanism in TRDM (Fig. 6a). Indeed, previous genome-wide analysis of 5-hydroxymethylcytosine uncovered only minor contribution of this modification to the epigenetic landscape in MPI [50]. However, in our data, DNA demethylation in at the P-to-D transition in narrow genomic windows is consistent with active DNA methylation and corroborates the previous study [50]. Taken together, the above results strongly suggest that reduction of DNA methylation in TRDM occurs primarily by passive, DNA replication-coupled mechanism.

Gradual but uneven genome-wide DNA remethylation occurs over the period of 70-h spanning early MPI substages. Given the predominance of hemimethylated DNA in the meiotic genome, restoration of premeiotic levels of DNA methylation is likely accomplished by *Dnmt1* whose mRNA expression (along with *Uhrf1*) gradually recovers in P and D (Fig. 6a). In addition, despite low mRNA expression levels of de novo methyltransferases, we cannot exclude a role for Dnmt3a2 in remethylation of MPI genomes (Fig. 6c). Cumulatively, the results support the idea of the leading role of passive DNA demethylation and DNMT1-mediated DNA remethylation in TRDM.

## Discussion

In this study, we systematically examined genome-wide DNA methylation across all MPI substages in adult male mice. This analysis provided the first evidence of a genome-wide, transient reduction of DNA methylation at the onset of meiosis. The central implication of this work is that critical MPI events (homology search, chromosome pairing, meiotic DSB formation and repair) occur in the context of hemimethylated genomic DNA.

With respect to the mechanism of DNA demethylation in TRDM, our data are most consistent with a passive, DNA replication-coupled mechanism. We base this conclusion on observations of (a) the initial drop of DNA methylation in PL cells going through meiotic S phase, (b) the genome-wide scope of DNA demethylation, (c) the dynamics of DNA methylation levels of early- and late-replicating domains in PL and L, (d) low expression of genes implicated in active DNA methylation and (e) direct measurement of the dynamics of DNA hemimethylation of L1 elements which allowed us to assess the extent of DNA hemimethylation in thousands of locations throughout the genome. Results of this analysis strongly support the idea of passive DNA replication mechanism of DNA demethylation at the onset of meiosis.

Overall, our analysis provides strong evidence of genome-wide DNA methylation by a passive, DNA replication-dependent mechanism. It is important to note that while a theoretical maximum reduction of DNA methylation levels is 50% of the starting values, the lower observed percentage in PL (by ~ 13 percentage points) is consistent with the non-uniform dynamics of DNA demethylation across individual genomes (early and late replication domains) and a non-uniform PL germ cell population studied (at different times of S phase, e.g., early, mid and late) which together lead to the apparently higher DNA methylation levels.

The dynamics of genome-wide CpG methylation point to a robust reduction of DNA methylation in PL compared to Spg. Interestingly, high methylation levels across Spg chromosomes argue against a possibility of preexisting DNA methylation levels determining the timing of DNA replication along chromosomes. Instead, consistent with semiconservative mechanism of DNA replication, DNA methylation levels remained high in late-replicating domains in PL but dropped in L upon replication of these regions in late PL, but before the recovery of DNA methylation.

Our observations underscore the uniqueness of meiotic S phase whose significance goes beyond being simply the last round of DNA replication prior to meiosis. Previous studies in numerous plant, fungal and animal species documented the longer duration of the meiotic S phase [2, 3, 51–54]. In addition, several studies provided evidence linking the meiotic S phase to meiotic recombination [38, 55–58]. Our results suggest that meiotic DNA replication in the male mice is different from somatic cells in that it fails to restore premeiotic DNA methylation levels in a timely manner.

What role(s) does TRDM play in meiosis? We envision several possibilities. First, TRDM might be a by-product of genome-wide remodeling of structure of chromosomes during mitosis-to-meiosis transition. Incorporation of meiosis-specific cohesin complexes, meiotic DNA recombination machinery and other events may preclude or reduce the accessibility of newly replicated DNA to DNMTs and their accessory proteins. Therefore, the observed reduction of DNA methylation might be non-consequential to meiosis.

Second, alternatively or in parallel, lower DNA methylation of the genome may create permissive conditions for some aspect of meiosis. Prior studies in plants, fungi and animals demonstrated that the disruption of normal pattern of DNA methylation in meiosis influences the meiotic recombination landscape [10–12, 59]. Although these prior studies do not demonstrate that the reduction of DNA methylation is an absolute prerequisite for wild-type levels of meiotic recombination, our finding of TRDM demonstrates that reduced DNA methylation is a common feature both of male and female meiotic germ cells of mice.

Third, DNA replication-coupled mechanism of TRDM suggests potential for distinct epigenetic states of hemimethylated sister chromatids of meiotic chromosomes. The impact of this epigenetic asymmetry of genetically identical DNA sequences on meiosis remains to be understood. We speculate that the epigenetic asymmetry of sister chromatids may lead to differential expression of their gene content. Indeed, as a result of passive DNA demethylation, genetically identical alleles will exist in distinct hemimethylated states with one allele inheriting the methylated coding strand while the coding strand of the other having no methylation. In addition, hemimethylation might stimulate inter-sister chromatid recombination. This idea is supported by early studies in cultured mammalian somatic cells that implicated DNA hemimethylation in increased sister chromatid exchanges in mitosis [60, 61]. In addition, given the essential role of the mismatch repair pathway in meiosis, it is tempting to speculate that DNA hemimethylation may influence the usage of sister chromatids in MPI akin to methylation-directed mismatch repair system of *E. coli* [62].

Finally, our results of analysis of L1 DNA methylation, and L1 RNA and protein expression suggest that TRDM contributes to gamete quality control. We base this conclusion on the finding of the appearance of fully unmethylated L1 elements that accounted for 2% of obtained sequencing reads in PL. This finding is consistent with incomplete DNA methylation (presumably hemimethylation) of a population of L1 elements in spermatogonia. Prior studies in embryonic stem cells and somatic cells demonstrated the existence of L1 elements showing DNA hemimethylation that necessitate cooperativity between de novo and maintenance DNA methyltransferases [63, 64]. We suspect that hemimethylated L1 elements remain largely transcriptionally silent until the meiotic S phase when they become fully demethylated and expressed. While fully demethylated L1s are the minority of potentially active L1 elements, they still correspond to dozens of L1 elements that may be expressed. Intriguingly, a potential role for TRDM in gamete quality control parallels a previously described selective elimination of MPI fetal oocytes with excessive L1 levels during the evolutionarily conserved process of fetal oocyte attrition [65]. If this were the case, L1 elements may be contributing to gamete quality control in meiotic germ cells in both sexes.

## Conclusions

Our results suggest that chromosomes exhibit dynamic changes in DNA methylation in MPI in male mice. These changes in DNA methylation arise by a passive mechanism during meiotic S phase resulting in hemimethylation of the genome in early MPI. We propose that TRDM facilitates meiotic prophase processes and gamete quality control.

## Methods

### Animals

Adult C57BL/6 J male mice (2- to 5-month-old) (Jackson Laboratory) were used as a source of adult testes. All experimental procedures were performed in compliance with ethical regulations and approved by the IACUC of Carnegie Institution for Science.

### Germ cell isolation

Germ cell fractions were enriched by fluorescence-activated cell sorting (FACS) as described previously [29]. Sorted germ cell fractions were devoid of somatic contamination but contained small amounts of germ cells from adjacent MPI stages. Cell fraction purity was determined to be > 85% for Spg, ~ 85% for PL, ~ 85% for L, ~ 80% for Z, > 90% for P, > 90% for D.

### Cryosections

After dissection of the testis, the tunica was removed, the testis was fixed (2% PFA in PBS) at 4C for 4 h, shaking. Samples were passed through sucrose solutions (10% for 1 h, 20% for 1 h, 30% overnight at 4C), embedded in OCT and stored at − 80 °C. Sections of 10 μm were used for IF.

### Immunofluorescence

IF on testicular sections or meiotic spreads was performed as described before [29]. ImageJ was used for image analysis.

### Antibodies

The following primary antibodies were for immunofluorescence (IF): monoclonal anti-γH2AX (Mouse, 1 mg/ml, 1:1000, Millipore 05-636), polyclonal anti-Sycp3 (Rabbit, 1 mg/ml, Abcam, ab15092. IF: 1:500 dilution), polyclonal anti-ORF1p (Rabbit, 1 mg/ml, a kind gift from Dr. Martin. IF: 1:500 dilution), monoclonal anti-Dmrt1 (Mouse, 200 µg/ml, Santa-Cruz, sc-10222. IF: 1:200 dilution), polyclonal anti-Dmrt6 (Rabbit, a kind gift from Dr. Zarkower. IF: 1:200 dilution), monoclonal anti-sp56 (Mouse, Pierce, MA1-10866. IF: 1:750). The following secondary antibodies (2 mg/ml) were used in this study: donkey anti-rabbit Alexa Fluor 594, donkey anti-rabbit Alexa Fluor 488, donkey anti-mouse Alexa Fluor 488.

### EdU labeling

Adult mice 1–3 months old were injected with 12.5 μg/g of body weight EdU (0.5 mg/ml DMSO stock) dissolved in 200 μL water. Mice were killed 2 h after injection and processed for FACS or for cryosections as described above. EdU detection with Click-iT EdU Alexa Fluor Kit was performed as described in the manual (Invitrogen).

### Whole-genome bisulfite sequencing (WGBS)

Each biological replicate consisted of pooled cells from 2 to 3 different animals from different FACS procedures. For WGBS, two biological replicates (2×) were used for Spg, PL, L, Z, P, D and epididymal spermatozoa (Spz). Genomic DNA (gDNA) was prepared by incubating cells in tail lysis buffer with 5 µl of Proteinase K (Life Technologies, 20 mg/ml) at 55 °C for 2–3 h. At the end of lysis, 2 µl of linear acrylamide (Ambion, 5 mg/ml) was added to samples. DNA was extracted with phenol–chloroform–isoamyl alcohol (Life Technologies, #15593) using Phase Lock Gel (PLG) Light tubes (5 Prime). One microliter of RNase A (Thermo Scientific, #EN0531, 10 mg/ml) was added to the aqueous phase, and the samples were incubated at 37 °C for 30 min, transferred to a PLG tube and mixed with chloroform. DNA was precipitated using ethanol and quantified with Quant-iT PicoGreen reagent (Molecular Probes) using SpectraMax microplate spectrophotometer. Isolated mouse gDNA was spiked with approximately 0.1% unmethylated cl857 Sam7 Lambda DNA (Promega) and sheared to fragments with a range of 200–600 bp using a Covaris M220 Ultrasonicator.

WGBS library preparation was based on Illumina’s ‘WGBS for Methylation Analysis’ protocol. The adaptor-ligated DNA fragments were processed for bisulfite conversion according to the manufacturer’s protocol (EZ DNA methylation Gold kit, Zymo Research). Bisulfite-treated DNA underwent 15 rounds of PCR amplification; libraries were prepared using Illumina *TruSeq* RNA Sample Prep Kit v2 and sequenced on Illumina HiSeq 2000 platform, yielding 100-bp paired-end reads. Each sample was run in a single lane, spiked with 5% Illumina PhiX genomic DNA control. Data were downloaded onto our servers in FASTQ format for processing.

### WGBS read alignment and extraction of methylation evidence

We used *Bismark* program for alignment of bisulfite-converted reads to mouse genome assembly NCBI37/mm9. The alignment was performed with respect to the bisulfite-treated Watson (original top) and Crick (original bottom) strands, and not their reverse complements, as the library was prepared in a strand-specific (directional) manner. No trimming was performed prior to alignment. After alignment *Bismark* de-duplication module was used to remove PCR duplicates.

*Bismark* was used to extract and summarize CpG methylation evidence present in the unique alignments. CpG evidence was filtered based on evaluation of methylation bias (M-bias) plots, and we excluded the first 6 nt from 5′ end of read 1 and 10 nt from read 2, and the last 1 nt from 3′ end of both reads prior to the extraction of methylation. Subsequently, using *Bismark*, we extracted CpG coverage into a file containing information for both strands. Finally, we merged strand-specific information. The final output text file contained chromosome (chr) name, chr start, chr end, CpG methylation percentage, count C and count unmethylated C. The final DNA methylation files were then examined with *bsseq* package and supplemented with R-based data analysis or in-house scripts.

### Bioinformatics analysis of global DNA methylation levels

Correlation between replicates of WGBS data was performed as follows: Biological replicates were compared pairwise (e.g., Spg1 with Spg2). Final *Bismark* output files containing CpG methylation and coverage were imported into R. DNA methylation was extracted and summarized in non-overlapping bins of 500 CpGs using *rep()* function, followed by aggregation of data and computation of mean values, using *aggregate()* function in R. Pearson correlation coefficient was then calculated using *cor()* function in R.

Global DNA methylation analysis was performed using *bsseq* package. Two replicate groups were formed, each consisted of seven samples (Spg, PL, L, Z, P, D and Spz) and made up a single *Bsseq* object. Only those CpGs that were covered by at least one read in all samples (common CpGs) were analyzed. Since only these common CpGs were involved in data analysis, the overall DNA methylation levels for each sample slightly differ from “raw” DNA methylation values obtained for all mappable CpGs of a given sample.

For plotting DNA methylation across the length of the chromosome, a custom Python script was used and DNA methylation was averaged using sliding non-overlapping windows of 100 kbp.

### Annotation used for DNA methylation analysis

The following genomic feature annotations were directly extracted from UCSC Table Browser based on mm9 genome: introns, exons, intergenic regions, CpG Islands and RepeatMasker (RMSK) repeats. Promoter coordinates were extracted from the UCSC/knownGene transcriptome file by taking + 1 kb to − 1 kb relative to the TSS, in a strand-conscious manner. The geneID and geneSymbols were obtained from specifying the selected fields in the output format and selecting knownGene (name), and kgXref (geneSymbol) fields. A custom script was used to change ucsc_ids to geneSymbols in the BED files. The genomic coordinates for imprinted gametic DMRs included 11 maternal (*Grb10*, *Igf2r*, *Impact*, *Kcnq1ot1*, *Mest*, *Nespas*-*Gnasxl*, *Peg10*, *Peg3*, *Snrpn*, *U2af1*-*rs1* and *Zac1*) and 3 paternal (*H19*, *Dlk1*-*Gt12* and *Rasgrf1*) DMRs.

### Analysis of sequencing coverage after WGBS

For chromosome-based visualization, de-duplicated BAM files were sorted using SAMtools (samtools.sourceforge.net). Individual chromosomes were analyzed separately (extracted from sorted BAM files). Biological replicates were analyzed separately for independent assessment. The files were uploaded into SeqMonk and read coverage was quantitated in the following manner: running, non-overlapping window probes of 5 kb were created to span the chromosome length. Read counts (the probes) were quantitated using the SeqMonk’s *Read Count Quantitation* approach where we counted all reads and corrected for total read count based on the largest data set. For overall coverage quantitation, running, non-overlapping window probes of 5 kb were created to span the chromosome length. Data store summary report was exported.

### Determination of overlap between datasets

Generally, overlaps were computed using *bedtools intersect*. For example, for intersecting DNA methylation with late-replicating regions, <intersect –wa –wb> was used, with replication timing (RT) file (-a) and DNA methylation file (-b). The RT file contained <chr RT/start RT/end RT/RT value>. The CpG methylation file contained <chr C/start C/end C/methylation level. Pearson correlation between DNA methylation values and RT values was performed using *cor()* function in R. To examine proportion of Spg-to-PL DMRs that overlap with PL-to-L and L-to-Z DMRs, we used bedtools intersect –wa –wb –a option to intersect BED files of DMRs in question.

### Analysis and annotation of differentially methylated regions (DMRs)

DMR analysis was performed using *Bsseq* package with previously optimized settings for DMR blocks. *Bsseq* employs local likelihood method, aggregating information from neighboring CpGs in a coverage-conscious manner and uses the combined data from two biological replicates to estimate DNA methylation at single CpG level. For this analysis, we required that each CpG be covered at least once in all four samples compared pairwise (two biological replicates per two stages). This selection resulted in a median CpG coverage of 3X–7X per sample (or 6X–14X per duplicate) and an overall coverage of > 77% of all genomic CpGs.

For analysis of DMRs and replication timing, genomic coordinates of DMR blocks were intersected with early or late replication timing (RT) coordinates. For every region within A (early or late RT domain), a number of intersections with B (DMR block) were computed. To calculate the proportion of an overlap between DMR blocks formed between Spg and PL (‘WTSpgPL’ DMRs) and all other DMR blocks, <*bedtools intersect* –wa –wb> was utilized, using a file containing WTSpgPL DMRs as (-a) and separate files, each containing DMRs between WT PL and L, L and Z, Z and P, P and D as (-b). Subsequent processing of the output involved adding the number of all intersections that matched WTSpgPL DMRs and normalizing to the total number of DMRs for a particular pairwise comparison.

Annotation from Illumina’s iGenomes (genes.gtf), based on the RefSeq dataset (July 2015), was used for annotating DMRs with genes.

We used Fisher’s exact test to examine the strength of overlap between DMRs and gene transcription. For each set of DMRs, a custom Python script was used to form a 2 × 2 table containing significantly upregulated genes that overlapped with DMRs, significantly upregulated genes that fell outside of DMRs, all genes found inside of the DMRs, and all genes found outside of the DMRs. To evaluate the significance of overlap, we calculated p values using Fisher’s exact test.

### Hairpin-bisulfite sequencing

Each sample consisted of pooled cells from up to two different animals from different FACS procedures. Sixty to 200 ng of DNA was restriction digested with BspE1 for 16 h at 37 °C and enzyme heat inactivated at 80 °C for 20 min. Digested DNA was extracted using phenol–chloroform–isoamyl, precipitated with ethanol and digestion verified on a 1% agarose gel. Genomic DNA was digested with BspEI, and the two complementary DNA strands were linked with a hairpin linker (5′P-CCGGGGGCCTATATAGTATAGGCCC) in a 25-μl reaction containing: 17 μl digest, 2.5 μl water, 2 μl 10 μM hairpin linker, 2.5 μl 10X T4 ligase reaction buffer and 1 μl (400U) of T4 ligase. The ligation reaction proceeded 10 h at 16C followed by bisulfite conversion using EZ DNA Methylation-Direct Kit (Zymo Research). Conditions for bisulfite conversion of hairpin L1 sequences were adjusted to include additional thermal denaturation steps (Laird et al., PNAS 2004) as follows: (1) 99 °C for 15 min, (2) 64 °C for 1 h, (3) 99 °C for 5 min, (4) 64 °C for 1.5 h, (5) 99 °C for 5 min, (6) 64 °C for 1.5 h. L1MdTf-specific PCR was performed in a 50-μl reaction containing 5 μl DNA, 1x PfU Turbo Cx reaction buffer and 2.5 units of Pfu Turbo Cx Hotstart DNA Polymerase (Agilent), 12.5 mM dNTPs, 10 μM each forward and reverse primers 5′TGGTAGTTTTTAGGTGGTATAGAT and 5′TCAAACACTATATTACTTTAACAATTCCCA resulting in 332-bp amplicon. PCR conditions were as follows: (1) 96 °C for 5 min followed by 35 cycles of (2) 96 °C for 60 s, (3) 55 °C for 45 s, (4) 72 °C for 90 s followed by the final extension at 72 °C for 5 min. PCR products were analyzed by agarose gel electrophoresis prior to processing for sequencing.

The resulting product was prepared for sequencing using Illumina *TruSeq* mRNA v2 kit, starting with end repair step, and sequenced on NextSeq 500. The 150 paired-end reads were aligned to L1MdTf promoter consensus sequence.

### Hairpin-bisulfite sequencing analysis

Fastq reads were trimmed with Trim Galore! using the following parameters: q 30, length 100, phred33, paired, three_prime_clip_R1 6, three_prime_clip_R2 6, stringency 5. We used *Bismark* [66] to align Reads 1 and 2 independently to Bismark-indexed L1TfMd 5′ end consensus sequence (5′ tccggaccggaggacaggtgcccacccggctggggaggcggcctaagccacagcagcagcggtcgccatcttggtcccgggactccaaggaacttaggaatttagtctgcttaagtgagagtctgtaccacctgggaactgccaaagcaacacagtgtctgagaaaggtcctgttttgg), using Bowtie 1 option. We used Bismak to align trimmed reads 1 and 2 to L1TfMd, independently, using the following parameters: non_directional, n 3, l 20. Subsequently, the reads were split into reads aligned to original bottom (OB) and those that aligned to complementary to original top (CTOT) using SAMtools. Thus, each read resulted in 2 files containing alignments to OB and CTOT, with read 1 OB plus read 2 CTOT, or read 2 OB plus read 1 CTOT corresponding to the two complementary strands of hairpin-bisulfite L1TfMd DNA. Bismark was used to extract CpG methylation from each file, and a custom script was used to generate a matrix where each line represented a “stiched” string of CpG methylation values for both strands of the hairpin-bisulfite L1TfMd DNA. Finally, R script was used to evaluate methylation, hemimethylation and unmethylated status of DNA.

### RNA-Seq

Total RNA was isolated from FACS-enriched fractions from adult C57BL6 male mouse testis. In most cases, due to the limited availability of enriched cells, total RNA from 2–4 mice (2–4 independent FACS enrichment sessions) was pooled to create one sample. One microliter of RNA was used for evaluation on the BioAnalyzer. Ribosomal RNA (rRNA) was removed from total RNA (up to 50 ng) using Ribo-Zero Gold rRNA Removal Kit according to the manufacturer’s protocol. The TruSeq RNA Sample Preparation Kit v2 was used to prepare cDNA library from ribosomal RNA-depleted RNA. The libraries were prepared as described in the manufacturer’s protocol (Pub. Part No.: 15026495) following low sample protocol. DNA fragments were enriched with PCR for 15 cycles. One microliter of the resulting library was used for validation and quantification analysis, using Agilent Technologies 2100 Bioanalyzer and Agilent DNA-1000 chip. The cDNA libraries were sequenced as single end 50-mers using the Illumina HiSeq 2000 platform, yielding a total of ~ 246 million reads (26–66 million total reads per sample).

The quality of the raw RNA-seq libraries was evaluated using fastQC (http://www.bioinformatics.babraham.ac.uk/projects/fastqc/). The fastQC-reported “Per base sequence quality” measure was very good, with more than 92% of all reads having a quality score of more than 30, and mean quality score of more than 36.

The read alignment was performed with TopHat (v2.0.7) [67], using short read mapping program Bowtie 2 (v2.0.6). During the alignment, we provided a transcriptome file that contained gene annotation. The reads were processed based on NCBI37/mm9 mouse genome and UCSC RefSeq gene annotation obtained from Illumina iGenomes (July 2015).

We used HTSeq package to count sequencing reads that overlap with gene transcriptome [68]. Specifically, we used <htseq-count –s no –a 10 input.sam iGenomes.gtf> command. The output is a tab-delimited text file containing counts for each gene (gene id and number of read counts). Subsequently, to evaluate differential expression we used edgeR. Specifically, we (1) built a counts table with all samples, using *DGElist* function, (2) normalized counts using the default TMM method, which accounts for compositional differences between the libraries, using *calcNormFactors* function, (3) obtained a table with normalized count-per-million (CPM), using *cpm* function, which we used directly for data analysis, or converted CPM to RPKM by (cpm/gene length/1000). For the differential expression analysis, an exact test was performed with an estimated Biological Coefficient of Variation (BCV) of 0.1, and *topTags* function was applied. A final table containing logFC (is log2FC), logCPM (is log2CPM), *p* value and FDR value for each gene was obtained.

For the analysis of the transcriptional landscape of repetitive elements, we used RepEnrich according to the suggested protocol [69]. Briefly, we aligned RNA-seq data to the genome using Bowtie 1 parameters that allow only unique mapping (-m1) and outputted multi-mapping and uniquely mapping reads into separate files. We ran RepEnrich python script on the data and then used EdgeR for subsequent processing of fraction counts file, which contained 1444 repetitive element entries. Specifically, we (1) built a counts table with all samples, using *DGElist* function, (2) normalized counts using the default TMM method, which accounts for compositional differences between the libraries, using *calcNormFactors* function, (3) obtained a table with normalized count-per-million (CPM), using *cpm* function, which we used directly for data analysis. For the differential expression analysis, an exact test was performed with an estimated dispersion specific to each pairwise comparison and *topTags* function was applied. A final table containing logFC (is log2FC), logCPM (is log2CPM), p value and FDR value for each repetitive element entry (subfamily) was obtained.

## Additional files

**Additional file 1: Figure S1.** Schematic representation of main events in meiotic prophase I. Following premeiotic DNA replication in preleptonema (PL), parental homologous chromosomes (each containing two sister chromatids) develop chromosome axes (marked by SYCP3 protein), pair and synapse in leptonema (L) and zygonema (Z). Synapse is complete in pachynema (P) indicated by the complete overlap of SYCP3 and SYCP1 proteins. Following the completion of meiotic recombination, the synaptonemal complex disassembles in diplonema (D). Approximate duration of MPI substages are indicated (hrs). Figure adapted from [70].

**Additional file 2: Figure S2.** Sample FACS analysis of adult murine testicular cells based on Hoechst 33342 dye staining. In summary, (A) initial gating on individual testicular populations based on Hoechst-blue and Hoechst-red fluorescence. R0 (excluded) is enriched in haploid spermatids; R1 (purple) is enriched in meiosis II spermatocytes; R2 (green) is enriched in leptotene (L); R3 is enriched in zygotene (Z) cells; R4 (dark green) is enriched in pachytene (P) spermatocytes; R5 (purple) is enriched in diplotene (D) spermatocytes; R6 (orange) contains spermatogonia (Spg) and somatic cells (Soma) that will be separated during subsequent back-gating; R7 (red) is enriched in preleptotene (PL) spermatocytes. (B) Gating tree formed after gating-based Hoechst dye staining followed by back-gating on forward scatter (FSC) and side scatter (SSC). Back-gating involves projection of a gate from the Hoechst plot onto an FSC/SSC plot, where the final Spg, L, Z, P and D enrichment gates are created. (C) Back-gates used to enrich for L (from R2 gate), Z (from R3 gate), P (from R4 gate) and D (from R5 gate). (D) Back-gates used to enrich for Spg and Soma (from R6 gate), shown in relation to P and D. (E) DNA content of enriched germ cells based on Hoechst-blue fluorescence histogram. The “DNA” gate used for cell sorting excludes 1C content (haploid) and includes cells with 2C through 4C DNA content where C is the amount of DNA within a haploid nucleus. The 2C region contains both, diploid Spg and Soma; the bimodal 4C region is enriched in L and Z and P and D spermatocytes; 2C-4C DNA content contains PL cells.

**Additional file 3: Figure S3.** Transcript abundance of select genes in individual MPI germ cells using RNA-seq, expressed as RPKM. Prominent transcripts from (A) testicular somatic cells, including Sertoli, Leydig and Macrophage cells, were examined to assess the level of contamination and included *Amh*, *Ccl2*, *Cd9*, *Cyp11a1*, *Cyp17a1*, *Fn1*, *Fshr*, *Gap43*, *Gata1*, *Gata4*, *Gpc3*, *Lhcgr*, *Lum*, *Mmp12*, *Mmp9*, *Pla2g4a*, *Rlf*, *Star*, *Tead2* and *Vcam1*. Meiosis-specific gene *Mlh3* is used for relative comparison. Transcript abundance of genes associated with (B) differentiated (blue) and undifferentiated (beige) Spg, (C) meiosis-specific synaptonemal complex (SC) formation, (D) meiotic onset (*Stra8*), meiosis-specific sister chromatid cohesion complex (*Smc1b*, *Rec8*, *Rad21l* and *Stag3*), recognition of meiotically programmed DSBs (*H2afx*) and other early meiosis-associated genes (e.g., *Mei1*), (E–F) DSB formation and repair and recombination were evaluated. (G-H) Replication-dependent histone variant genes are highly expressed and enriched in PL spermatocytes. Twelve replication-dependent histone variant genes with high transcript abundance are shown. Selected are whose genes that are known to be highly enriched in early spermatocytes at 9-dpp testis, but not 2-dpp (gonocytes), 25-dpp (enriched in round spermatids) or 60-dpp (enriched in haploid cells). (I) Two genes, *H3f3a* and *H3f3b*, encoding replication-independent histone H3.3 were examined.

**Additional file 4: Table S1.** Bisulfite sequencing, alignment and read de-duplication results summary.

**Additional file 5: Table S2.** The efficiency of bisulfite conversion based on unmethylated lambda DNA.

**Additional file 6: Table S3.** Methylation evidence results for the uniquely aligned, de-duplicated and M-bias filtered reads.

**Additional file 7: Table S4.** Pearson correlation between biological replicates.

**Additional file 8: Table S5.** Coverage and methylation evidence for common CpGs (covered in common between samples of individual biological replicate groups).

**Additional file 9: Figure S4.** CpG DNA methylation levels across chromosome length. DNA methylation was averaged using sliding non-overlapping windows of 100 kb.

**Additional file 10: Figure S5.** CpG DNA methylation levels across chromosome length. DNA methylation was averaged using sliding non-overlapping windows of 100 kb.

**Additional file 11: Figure S6.** DNA methylation dynamics on chromosome X compared to autosomes. Biological replicate 1 (left panel) and 2 (right panel): were examined independently. CpG methylation was averaged in bins of 100 CpGs (A), and then, Pearson correlation was calculated (B). The number of different CpGs (CpG loci) evaluated for biological replicates 1 and 2 was as follows: chrX (214,981 and 266,439), chr1 (6,983,222 and 7,564,250), chr2 (1,022,879 and 1,103,479), chr3 (805,182 and 875,167), chr19 (383,441 and 412,451) all minus chrX (no chrX) (13,667,873 and 14,804,983).

**Additional file 12: Figure S7.** (A) Box-and-whisker plot of DNA methylation levels across promoters and CpG islands. The average DNA methylation levels were aggregated as consecutive, non-overlapping averages of 100 CpGs. Averages were combined for biological replicates. (B) Box-and-whisker plot of DNA methylation levels across various genomic features. The average DNA methylation levels were aggregated as consecutive, non-overlapping averages of 100 CpGs. Averages were combined for biological replicates. (C) Plots of mean DNA methylation levels of maternal and paternal select imprinted differentially methylated regions (DMRs) across MP.

**Additional file 13: Table S6.** Average DNA methylation results for different functional genomic elements.

**Additional file 14: Table S7.** Characteristics of pairwise comparisons for large DMR block analysis.

**Additional file 15: Table S8.** (A) Features of large differentially methylated blocks in germ cells. (B) Mean percentage (%) of genomic feature quantitation (covered by remethylating DMRs).

**Additional file 16: Figure S8.** DNA methylation pattern in PL overlaps with replication timing, an example of chromosome 14, biological replicate one. (A) Plot of CpG DNA methylation of MPI stages, premeiotic Spg and post-meiotic Spz, across chromosome 14, which is ~ 125 Mbp long. Biological Replicate 1 is shown. (B) Replication timing (RT) data from CH12 cells (mouse B cell lymphoma) [71] and (C) genome sequencing coverage after WGBS-seq, viewed in SeqMonk program.

**Additional file 17: Figure S9.** DNA methylation pattern in PL overlaps with replication timing, an example of chromosome 16. Biological replicate 1 is shown on the left, and replicate 2 on the right. (A,D) DNA methylation, (B,E) replication timing (RT) and (C, F) genome sequencing coverage for two biological replicates.

**Additional file 18: Figure S10.** Genome-wide relationship between replication timing and DNA methylation. (A) Replication timing (RT) data from CH12 cells (mouse B cell lymphoma) [71] were correlated with CpG DNA methylation corresponding to late RT domains. Note a prominent switch in correlation directionality from PL to L. Biological replicates (Reps 1 and 2) were processed individually and are shown in light and dark red. (B) The PL cell fraction enriched by FACS contains replicating cells. More than 70% of FACS-enriched PL cells are EdU + , enriched in mid- and late- S phase, based on the characteristic EdU staining patterns.

**Additional file 19: Figure S11.** L1 hairpin-bisulfite sequencing amplicon analysis. Specific primers, Primer 1 and Primer 2 were used to amplify bisulfite-converted (BSC) and hairpin-linked L1MdTf promoter region. Primer 1 corresponds to BSC original top (OT) strand; Primer 2 corresponds to BSC complementary to original top (CTOT) strand. CpGs analyzed for hemimethylation with hairpin-bisulfite sequencing are indicated.

**Additional file 20: Table S9.** Hairpin-bisulfite sequencing of L1, alignment results summary.

**Additional file 21: Figure S12.** Analysis of transcript abundance of repetitive elements by RNA-seq. RepEnrich (fractional counts strategy) was used to calculate the total abundance of repetitive elements, expressed in counts per million mapping reads (CPM) for A) reads that map to all types of repeats annotated by repeat masker (n = 1266 types) and B) reads that map to LINE subfamilies (n = 121)(Supplemental Table S11).

**Additional file 22: Figure S13.** L1ORF1p expression in MPI of male germ cells. Temporal expression of L1ORF1p (green) was evaluated in testicular cryosections in the context of seminiferous epithelial cycle composed of stages I–XII. Haploid spermatids are identified based on numbers 1 through 16 according to degree of differentiation (only some are highlighted here). The basal membrane is outlined by the bright cross-reacting red staining. Following spermatogenic progression based on acrosome development marked by sp56 (red) and DNA stain, DAPI (blue), it is determined that cytoplasmic L1ORF1p is first seen in L/Z spermatocytes at stage XI (or X–XI) and persists from Z (stages XI–XII) to mid-P spermatocytes (stages I through VI–VII). L1ORF1p is not detectable in late P cells found stages VII–X, but is evident in the cytoplasm of elongating spermatids (see stages VII–IX) and is also detected as small dots in early round spermatids. The sp56 staining for spermatids beyond step 13 is difficult to see here, since the acrosome spreads very thin at this time. The selection inside the white box of the merged image (top row) is shown as a close-up inset in the DAPI-containing image row and represents a single confocal plane in an otherwise 3-D stacked image, highlighting the cytoplasmic distribution of L1ORF1p in meiotic prophase I spermatocytes. Bar = 10 micron.
