# Supplementary figures and images for: Transient reduction of DNA methylation at the onset of meiosis in male mice
Source: Epigenetics Chromatin. 2018 Apr 4;11:15. doi: 10.1186/s13072-018-0186-0 (PMC5883305; doi:10.1186/s13072-018-0186-0)

**Fig. S1**

**MEIOTIC PROPHASE I**

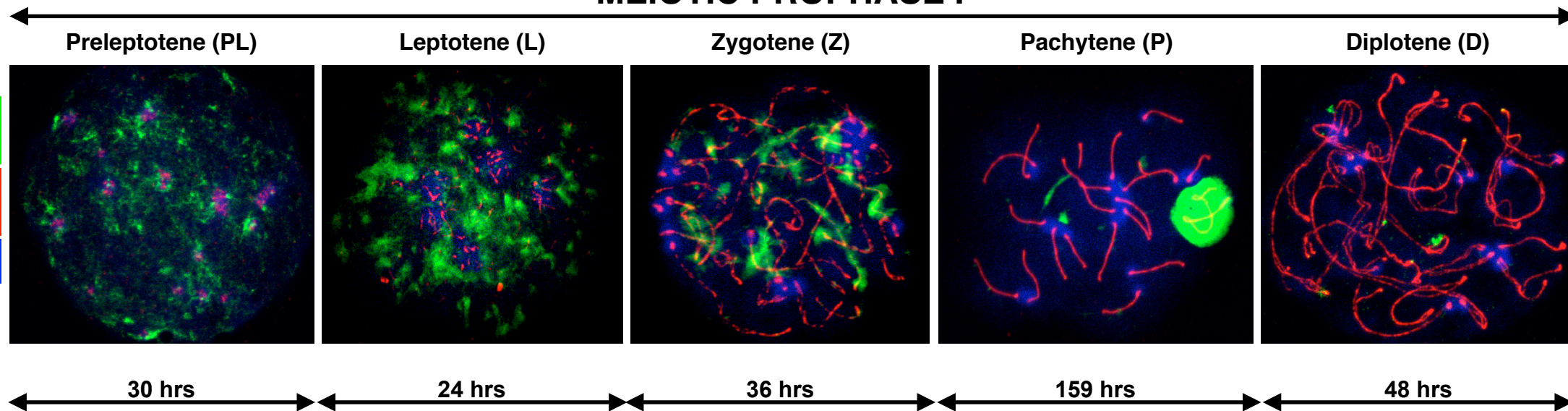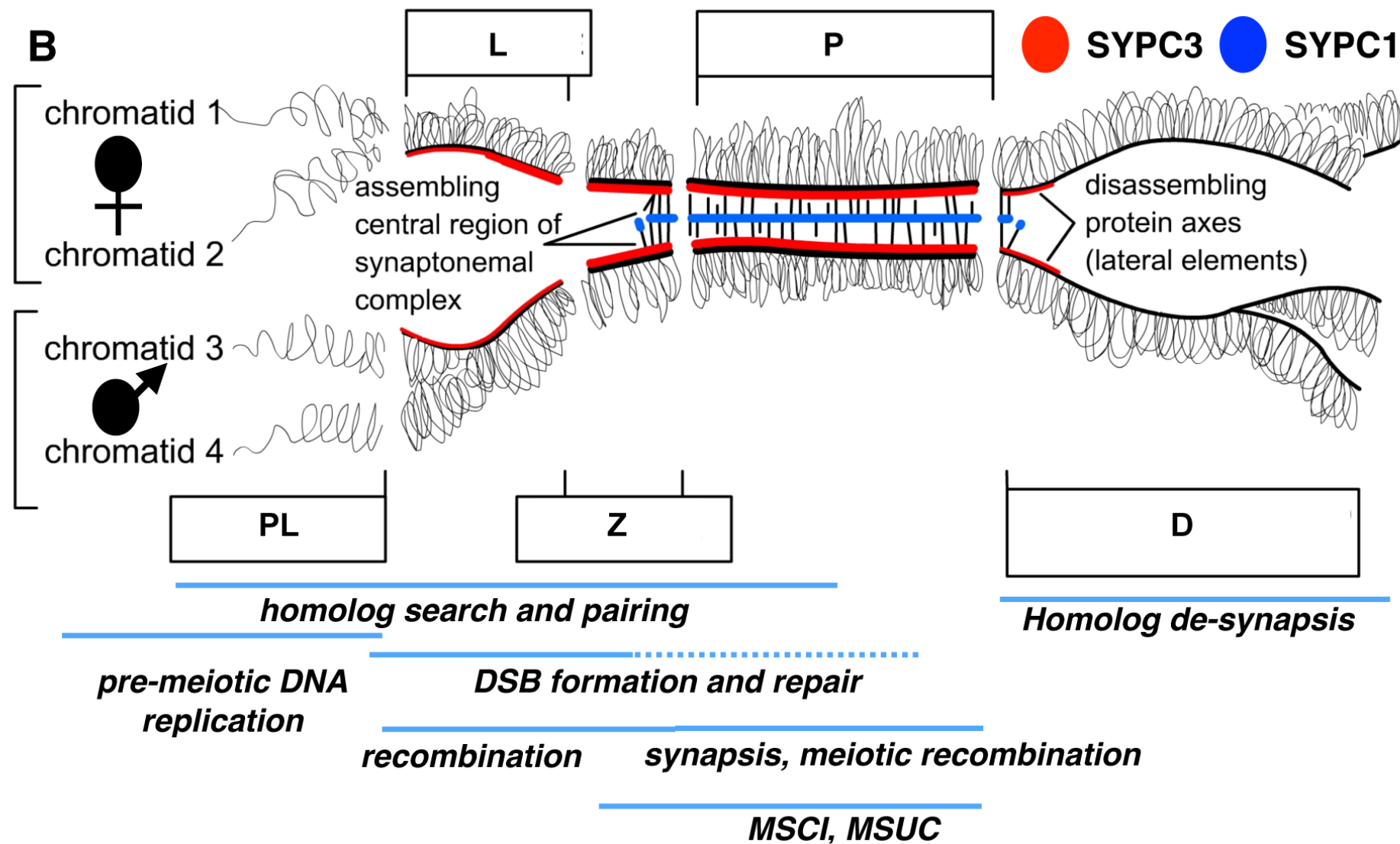

Supplement: Supplementary file 1 — Additional file 1: Figure S1. Schematic representation of main events in meiotic prophase I. Following premeiotic DNA replication in preleptonema (PL), parental homologous chromosomes (each containing two sister chromatids) develop chromosome axes (marked by SYCP3 protein), pair and synapse in leptonema (L) and zygonema (Z). Synapse is complete in pachynema (P) indicated by the complete overlap of SYCP3 and SYCP1 proteins. Following the completion of meiotic recombination, the synaptonemal complex disassembles in diplonema (D). Approximate duration of MPI substages are indicated (hrs). Figure adapted from [70]. [file 13072_2018_186_MOESM1_ESM.pdf]

**Fig. S2**

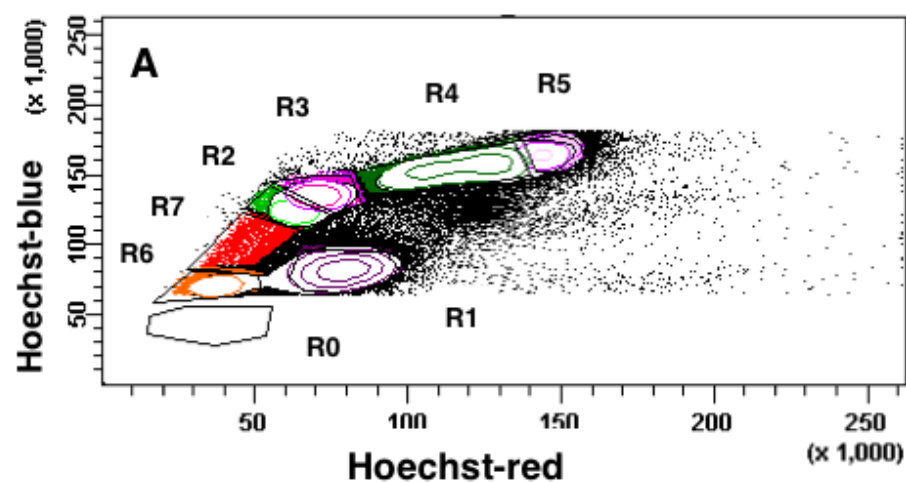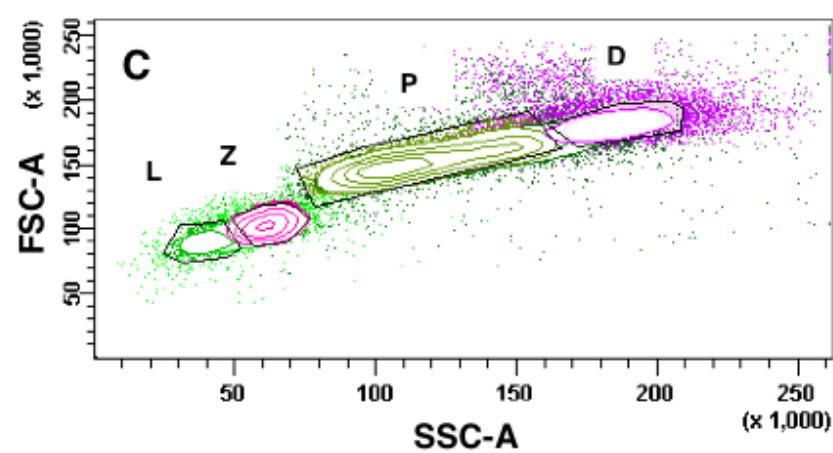

**B**

| Population | #Events   | %Parent | %Total |
|------------|-----------|---------|--------|
| All Events | 1,000,000 | ####    | 100.0  |
| Cells      | 500,947   | 50.1    | 50.1   |
| Alive      | 457,428   | 91.3    | 45.7   |
| DNA        | 407,699   | 89.1    | 40.8   |
| R0         | 312,057   | 76.5    | 31.2   |
| R1         | 21,765    | 5.3     | 2.2    |
| R2         | 4,554     | 1.1     | 0.5    |
| L          | 1,303     | 28.6    | 0.1    |
| R3         | 6,939     | 1.7     | 0.7    |
| Z          | 3,967     | 57.2    | 0.4    |
| R4         | 17,687    | 4.3     | 1.8    |
| P          | 13,620    | 77.0    | 1.4    |
| R5         | 5,344     | 1.3     | 0.5    |
| D          | 2,607     | 48.8    | 0.3    |
| R6         | 4,536     | 1.1     | 0.5    |
| Spq        | 1,707     | 37.6    | 0.2    |
| Soma       | 1,383     | 30.5    | 0.1    |
| R7         | 2,057     | 0.5     | 0.2    |

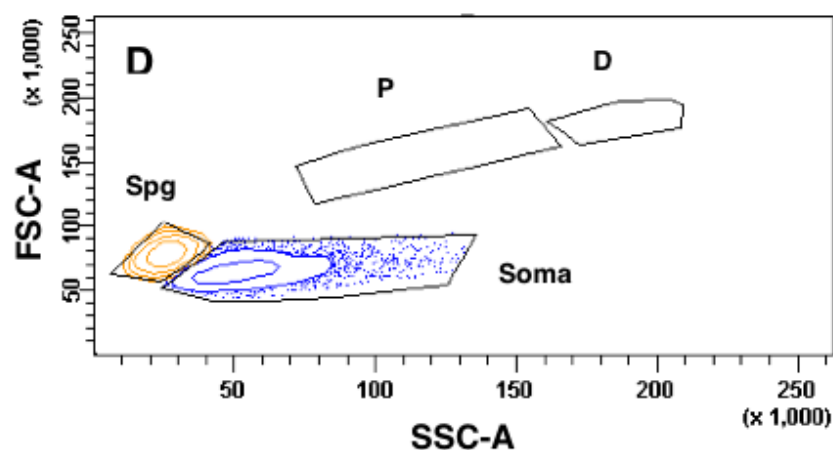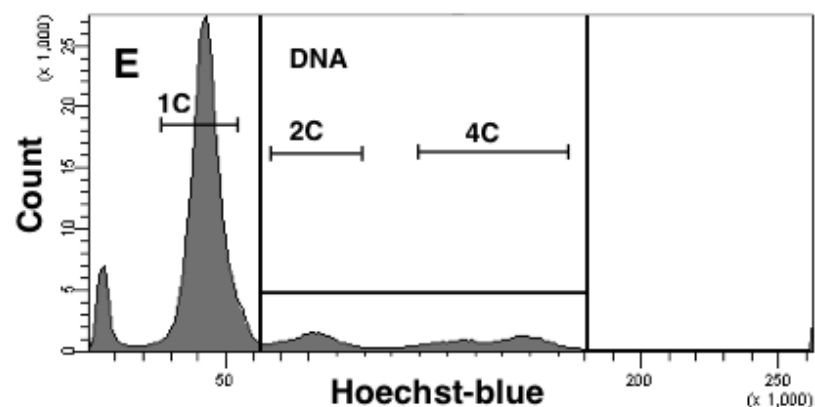

Supplement: Supplementary file 2 — Additional file 2: Figure S2. Sample FACS analysis of adult murine testicular cells based on Hoechst 33342 dye staining. In summary, (A) initial gating on individual testicular populations based on Hoechst-blue and Hoechst-red fluorescence. R0 (excluded) is enriched in haploid spermatids; R1 (purple) is enriched in meiosis II spermatocytes; R2 (green) is enriched in leptotene (L); R3 is enriched in zygotene (Z) cells; R4 (dark green) is enriched in pachytene (P) spermatocytes; R5 (purple) is enriched in diplotene (D) spermatocytes; R6 (orange) contains spermatogonia (Spg) and somatic cells (Soma) that will be separated during subsequent back-gating; R7 (red) is enriched in preleptotene (PL) spermatocytes. (B) Gating tree formed after gating-based Hoechst dye staining followed by back-gating on forward scatter (FSC) and side scatter (SSC). Back-gating involves projection of a gate from the Hoechst plot onto an FSC/SSC plot, where the final Spg, L, Z, P and D enrichment gates are created. (C) Back-gates used to enrich for L (from R2 gate), Z (from R3 gate), P (from R4 gate) and D (from R5 gate). (D) Back-gates used to enrich for Spg and Soma (from R6 gate), shown in relation to P and D. (E) DNA content of enriched germ cells based on Hoechst-blue fluorescence histogram. The “DNA” gate used for cell sorting excludes 1C content (haploid) and includes cells with 2C through 4C DNA content where C is the amount of DNA within a haploid nucleus. The 2C region contains both, diploid Spg and Soma; the bimodal 4C region is enriched in L and Z and P and D spermatocytes; 2C-4C DNA content contains PL cells. [file 13072_2018_186_MOESM2_ESM.pdf]

**Fig. S3**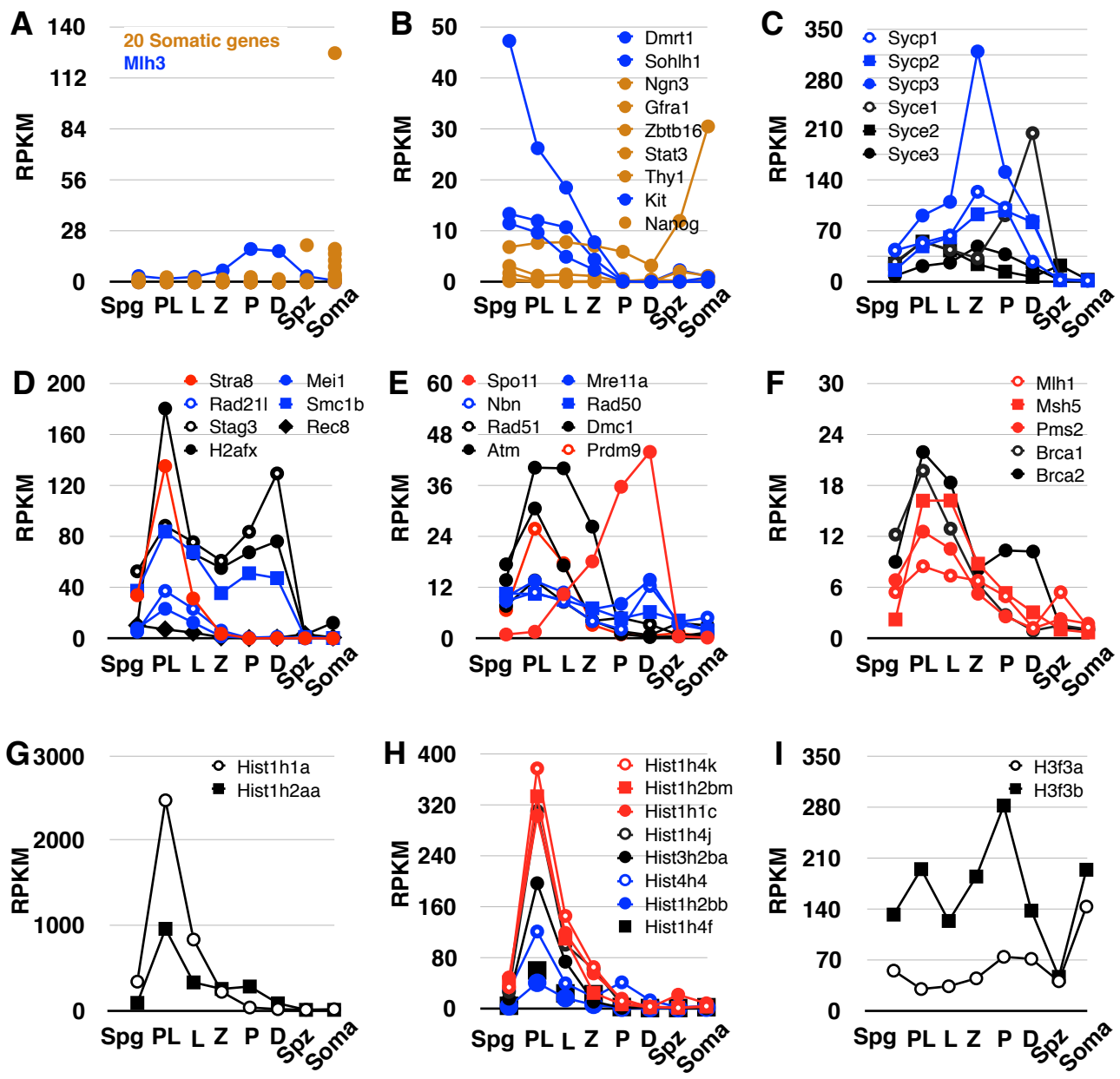

Supplement: Supplementary file 3 — Additional file 3: Figure S3. Transcript abundance of select genes in individual MPI germ cells using RNA-seq, expressed as RPKM. Prominent transcripts from (A) testicular somatic cells, including Sertoli, Leydig and Macrophage cells, were examined to assess the level of contamination and included Amh, Ccl2, Cd9, Cyp11a1, Cyp17a1, Fn1, Fshr, Gap43, Gata1, Gata4, Gpc3, Lhcgr, Lum, Mmp12, Mmp9, Pla2g4a, Rlf, Star, Tead2 and Vcam1. Meiosis-specific gene Mlh3 is used for relative comparison. Transcript abundance of genes associated with (B) differentiated (blue) and undifferentiated (beige) Spg, (C) meiosis-specific synaptonemal complex (SC) formation, (D) meiotic onset (Stra8), meiosis-specific sister chromatid cohesion complex (Smc1b, Rec8, Rad21l and Stag3), recognition of meiotically programmed DSBs (H2afx) and other early meiosis-associated genes (e.g., Mei1), (E–F) DSB formation and repair and recombination were evaluated. (G-H) Replication-dependent histone variant genes are highly expressed and enriched in PL spermatocytes. Twelve replication-dependent histone variant genes with high transcript abundance are shown. Selected are whose genes that are known to be highly enriched in early spermatocytes at 9-dpp testis, but not 2-dpp (gonocytes), 25-dpp (enriched in round spermatids) or 60-dpp (enriched in haploid cells). (I) Two genes, H3f3a and H3f3b, encoding replication-independent histone H3.3 were examined. [file 13072_2018_186_MOESM3_ESM.pdf]

**Fig. S4**

**Replicate 1**

**Mean CpG Methylation**

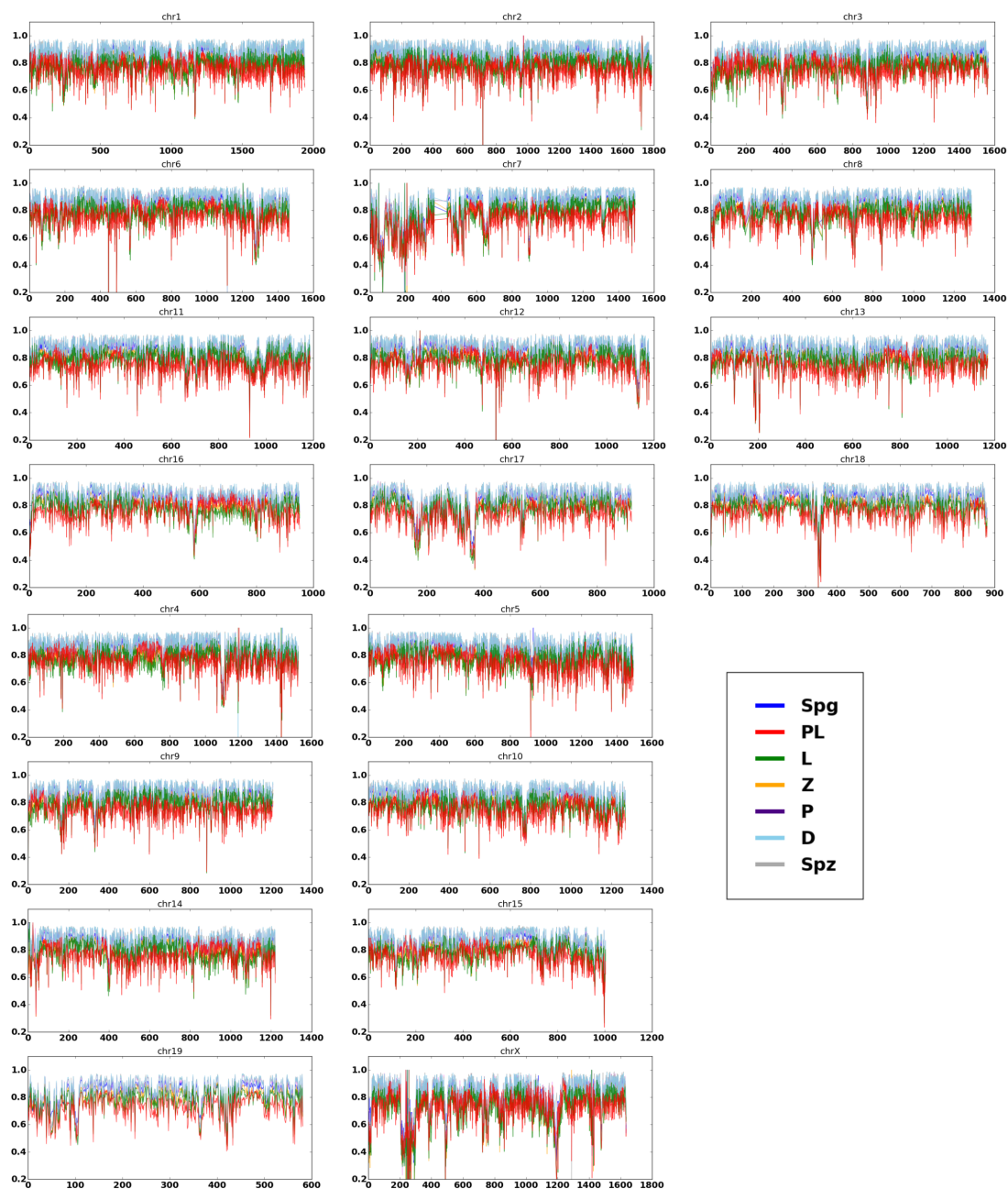

**CpG position across chromosome (100 kb bins)**

Supplement: Supplementary file 9 — Additional file 9: Figure S4. CpG DNA methylation levels across chromosome length. DNA methylation was averaged using sliding non-overlapping windows of 100 kb. [file 13072_2018_186_MOESM9_ESM.pdf]

**Fig. S5**

**Replicate 2**

**Mean CpG Methylation**

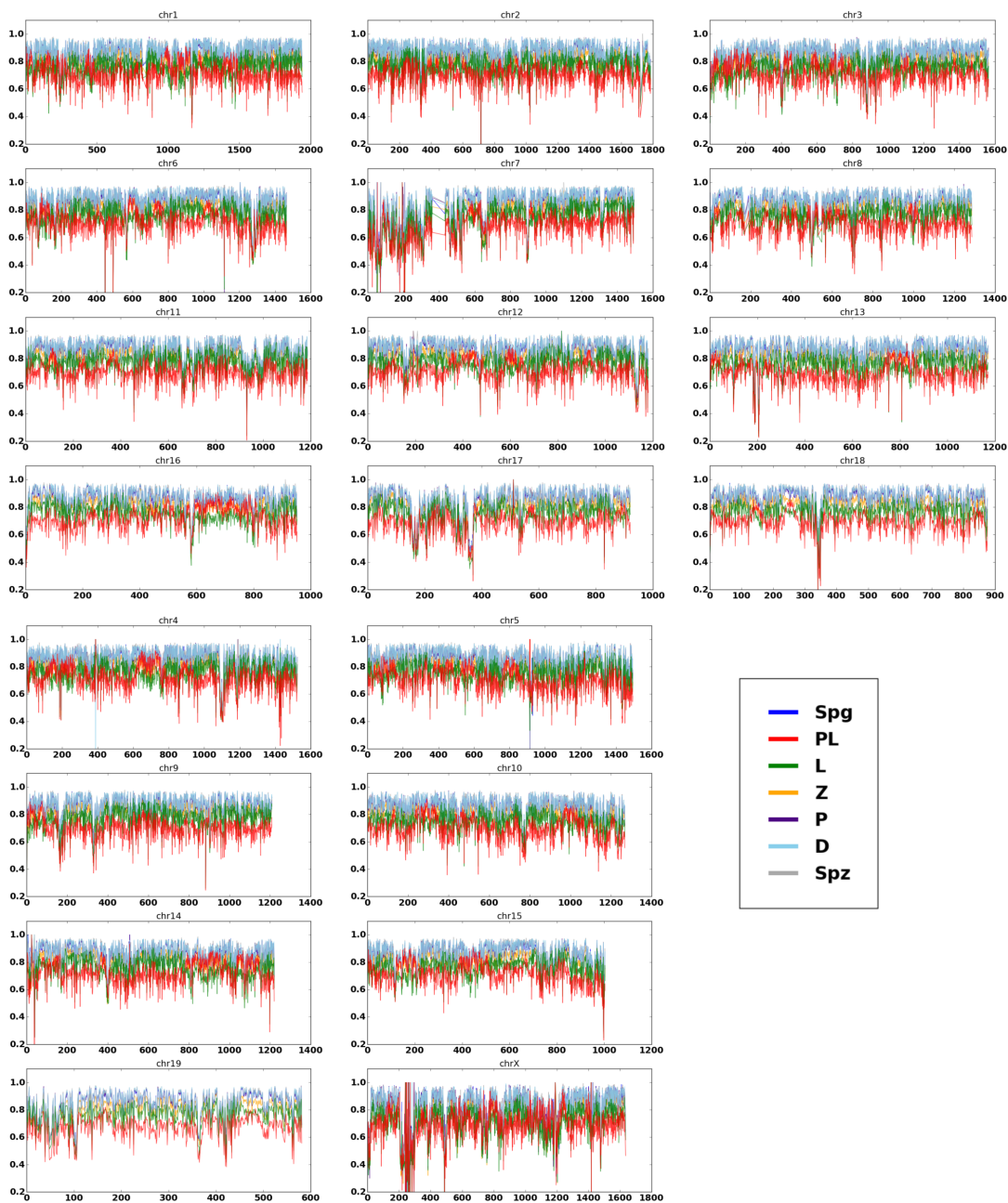

**CpG position across chromosome (100 kb bins)**

Supplement: Supplementary file 10 — Additional file 10: Figure S5. CpG DNA methylation levels across chromosome length. DNA methylation was averaged using sliding non-overlapping windows of 100 kb. [file 13072_2018_186_MOESM10_ESM.pdf]

**Fig. S6**

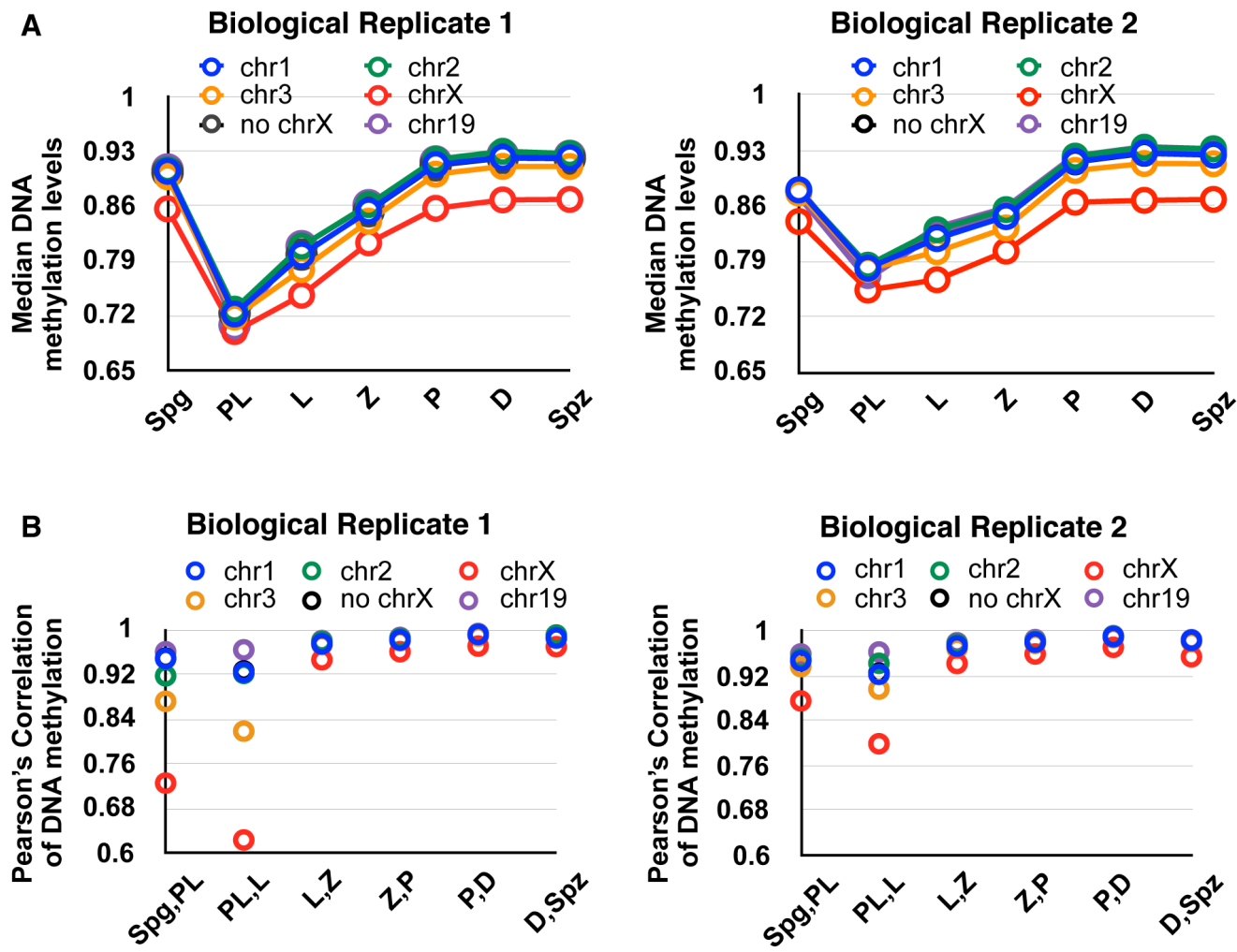

Supplement: Supplementary file 11 — Additional file 11: Figure S6. DNA methylation dynamics on chromosome X compared to autosomes. Biological replicate 1 (left panel) and 2 (right panel): were examined independently. CpG methylation was averaged in bins of 100 CpGs (A), and then, Pearson correlation was calculated (B). The number of different CpGs (CpG loci) evaluated for biological replicates 1 and 2 was as follows: chrX (214,981 and 266,439), chr1 (6,983,222 and 7,564,250), chr2 (1,022,879 and 1,103,479), chr3 (805,182 and 875,167), chr19 (383,441 and 412,451) all minus chrX (no chrX) (13,667,873 and 14,804,983). [file 13072_2018_186_MOESM11_ESM.pdf]

**Fig. S7****A**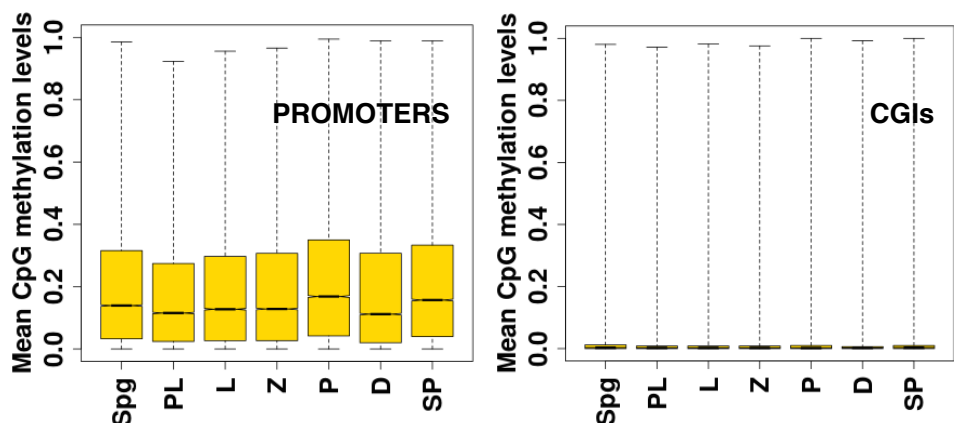**B**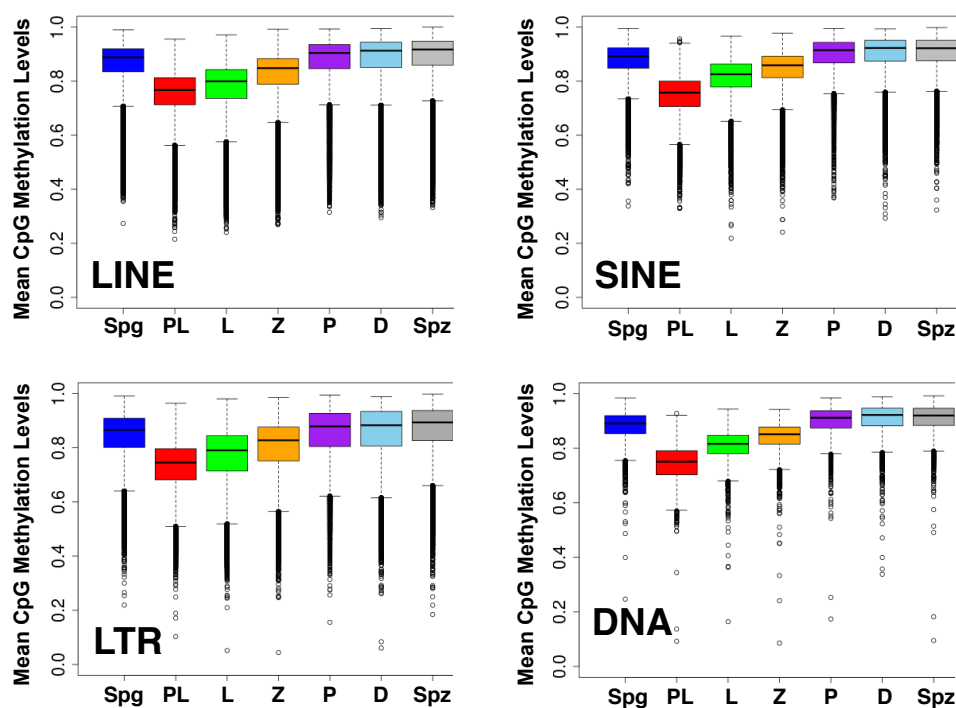**C**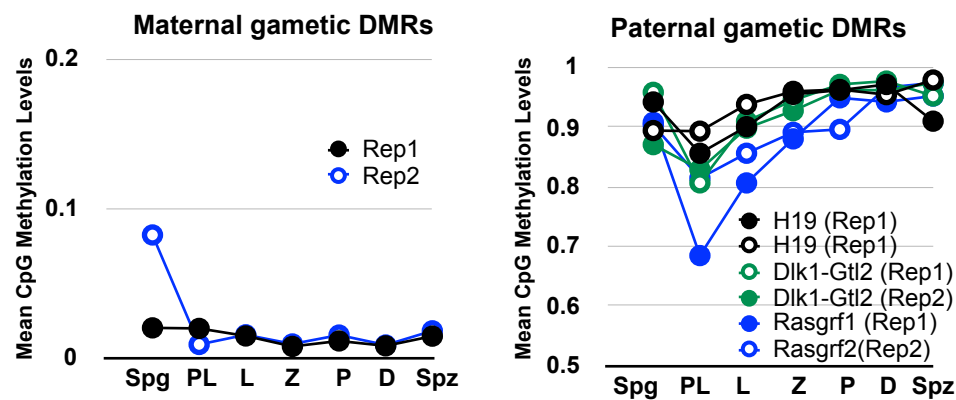

Supplement: Supplementary file 12 — Additional file 12: Figure S7. (A) Box-and-whisker plot of DNA methylation levels across promoters and CpG islands. The average DNA methylation levels were aggregated as consecutive, non-overlapping averages of 100 CpGs. Averages were combined for biological replicates. (B) Box-and-whisker plot of DNA methylation levels across various genomic features. The average DNA methylation levels were aggregated as consecutive, non-overlapping averages of 100 CpGs. Averages were combined for biological replicates. (C) Plots of mean DNA methylation levels of maternal and paternal select imprinted differentially methylated regions (DMRs) across MP. [file 13072_2018_186_MOESM12_ESM.pdf]

**Fig. S8**

**A**

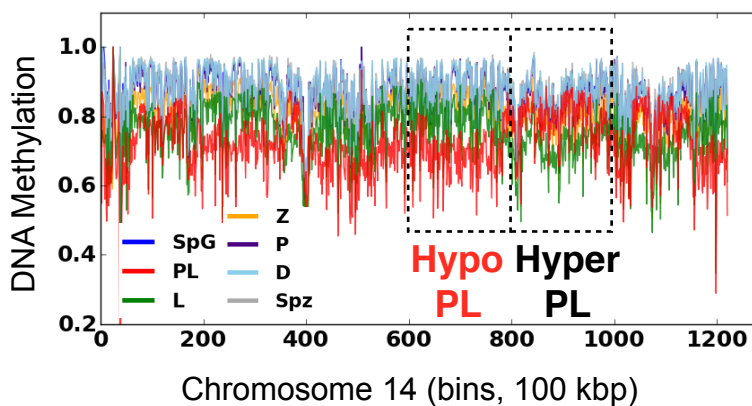

**B**

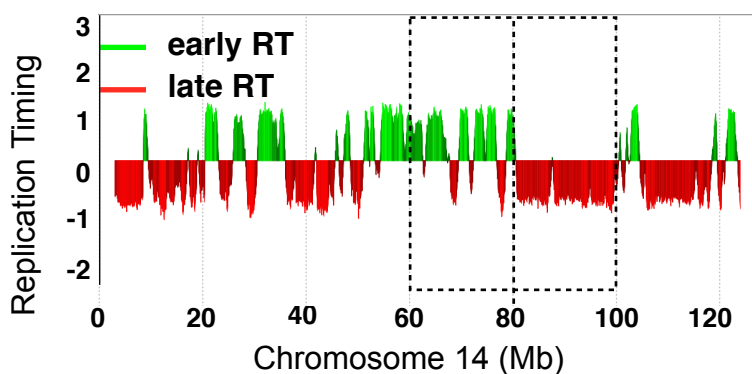

**C**

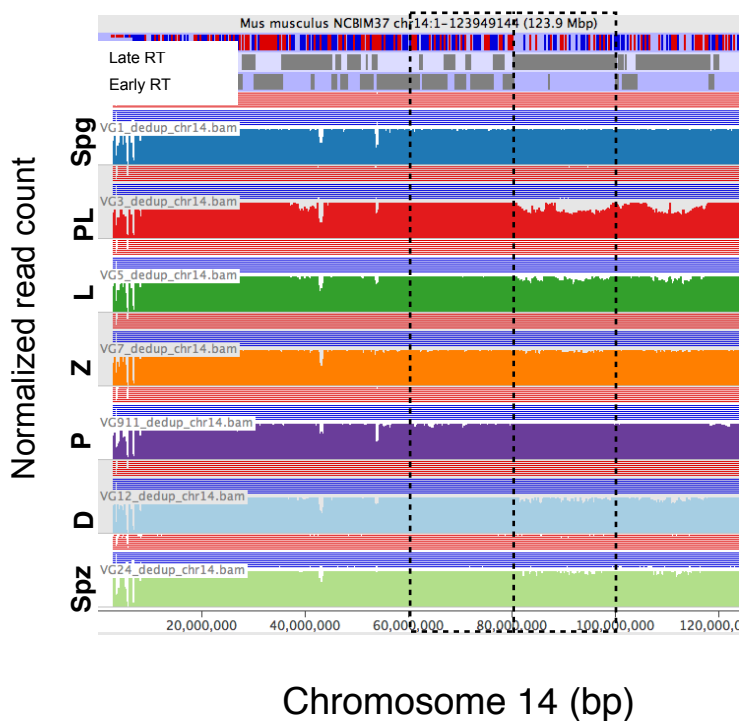

Supplement: Supplementary file 16 — Additional file 16: Figure S8. DNA methylation pattern in PL overlaps with replication timing, an example of chromosome 14, biological replicate one. (A) Plot of CpG DNA methylation of MPI stages, premeiotic Spg and post-meiotic Spz, across chromosome 14, which is ~ 125 Mbp long. Biological Replicate 1 is shown. (B) Replication timing (RT) data from CH12 cells (mouse B cell lymphoma) [71] and (C) genome sequencing coverage after WGBS-seq, viewed in SeqMonk program. [file 13072_2018_186_MOESM16_ESM.pdf]

**Fig. S9**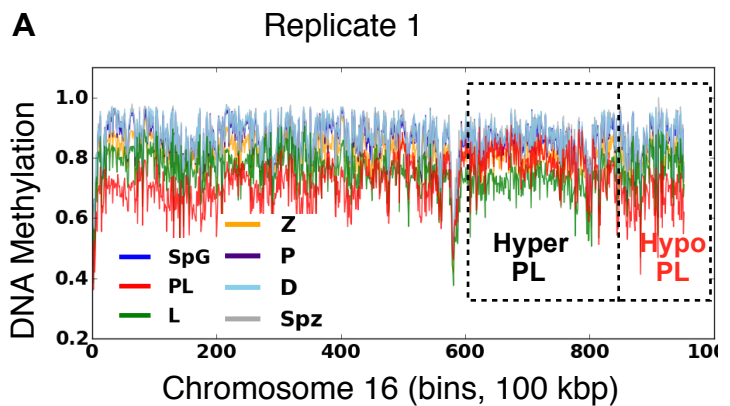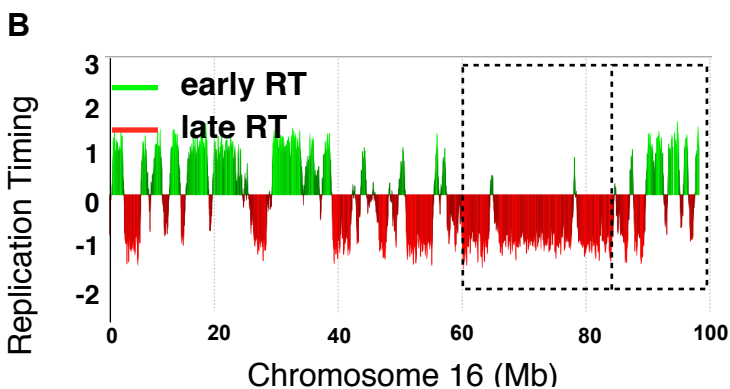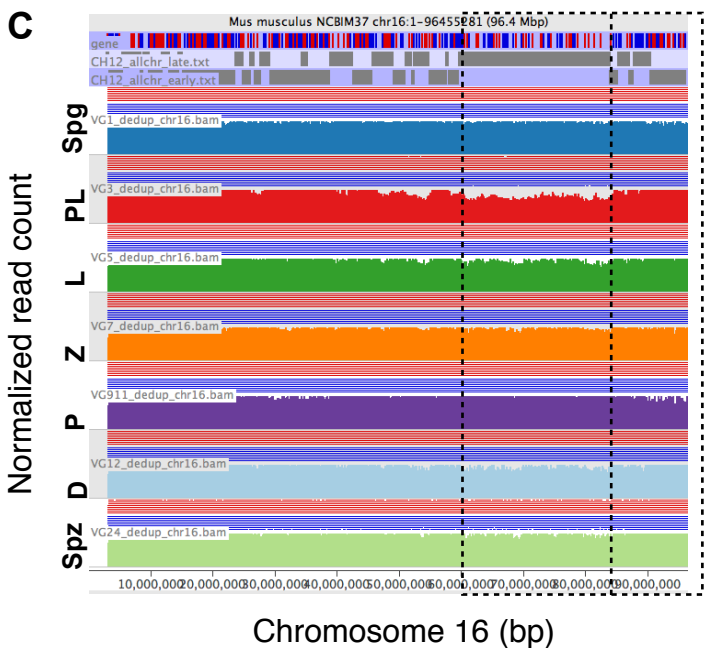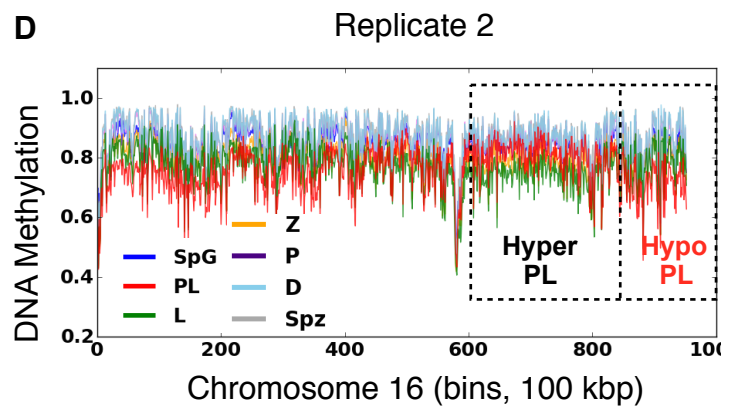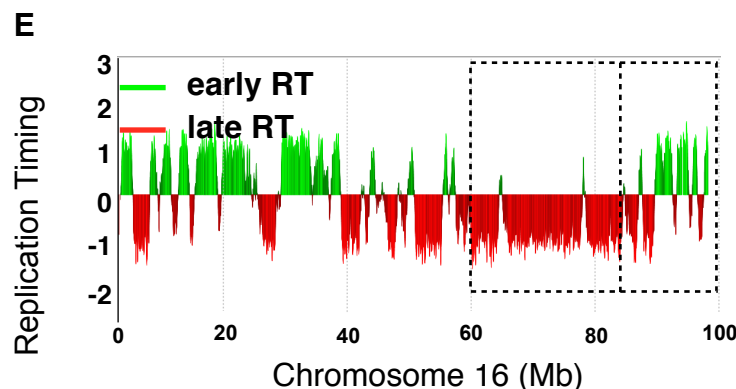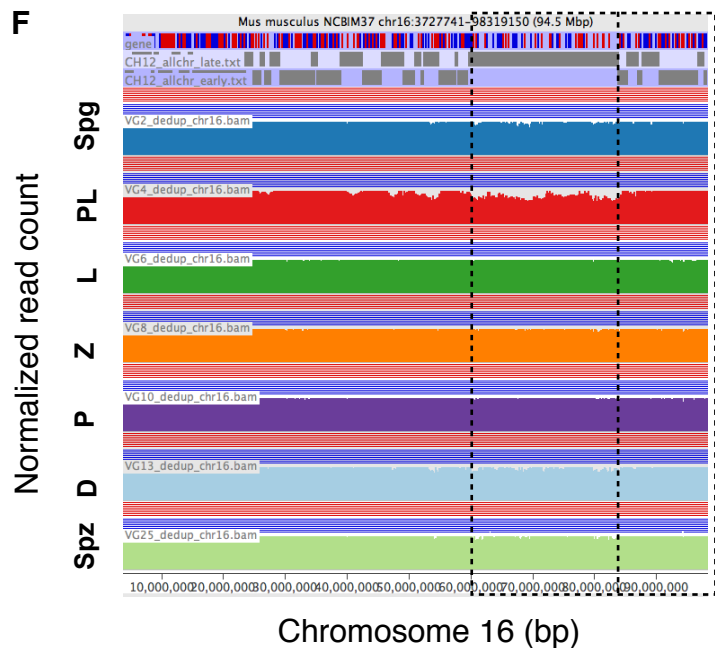

Supplement: Supplementary file 17 — Additional file 17: Figure S9. DNA methylation pattern in PL overlaps with replication timing, an example of chromosome 16. Biological replicate 1 is shown on the left, and replicate 2 on the right. (A,D) DNA methylation, (B,E) replication timing (RT) and (C, F) genome sequencing coverage for two biological replicates. [file 13072_2018_186_MOESM17_ESM.pdf]

**Fig. S10**

**A**

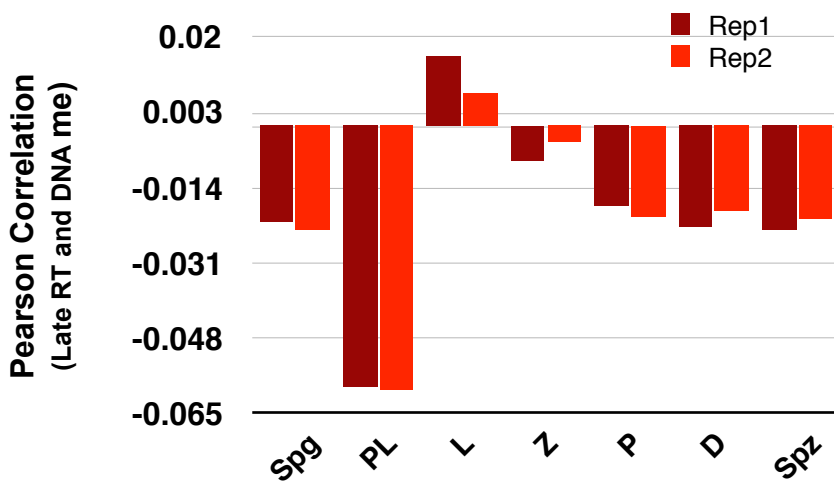

**B**

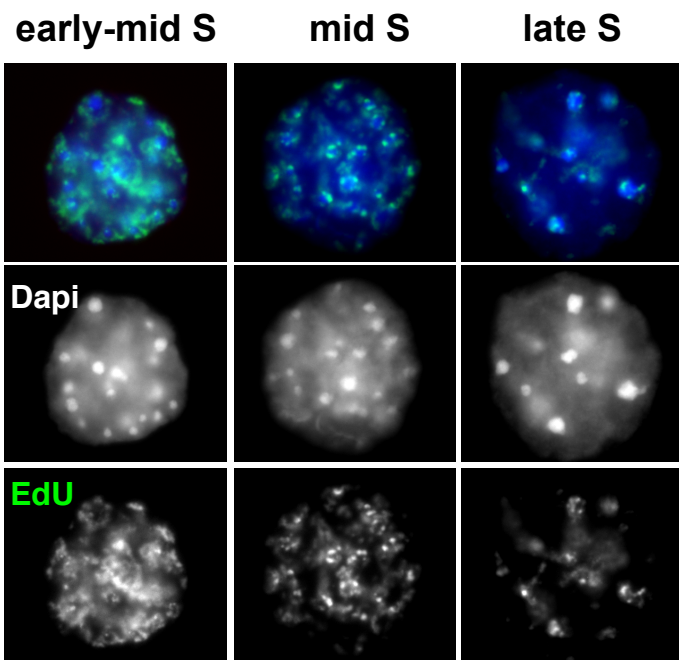

Supplement: Supplementary file 18 — Additional file 18: Figure S10. Genome-wide relationship between replication timing and DNA methylation. (A) Replication timing (RT) data from CH12 cells (mouse B cell lymphoma) [71] were correlated with CpG DNA methylation corresponding to late RT domains. Note a prominent switch in correlation directionality from PL to L. Biological replicates (Reps 1 and 2) were processed individually and are shown in light and dark red. (B) The PL cell fraction enriched by FACS contains replicating cells. More than 70% of FACS-enriched PL cells are EdU + , enriched in mid- and late- S phase, based on the characteristic EdU staining patterns. [file 13072_2018_186_MOESM18_ESM.pdf]

### Fig. S11

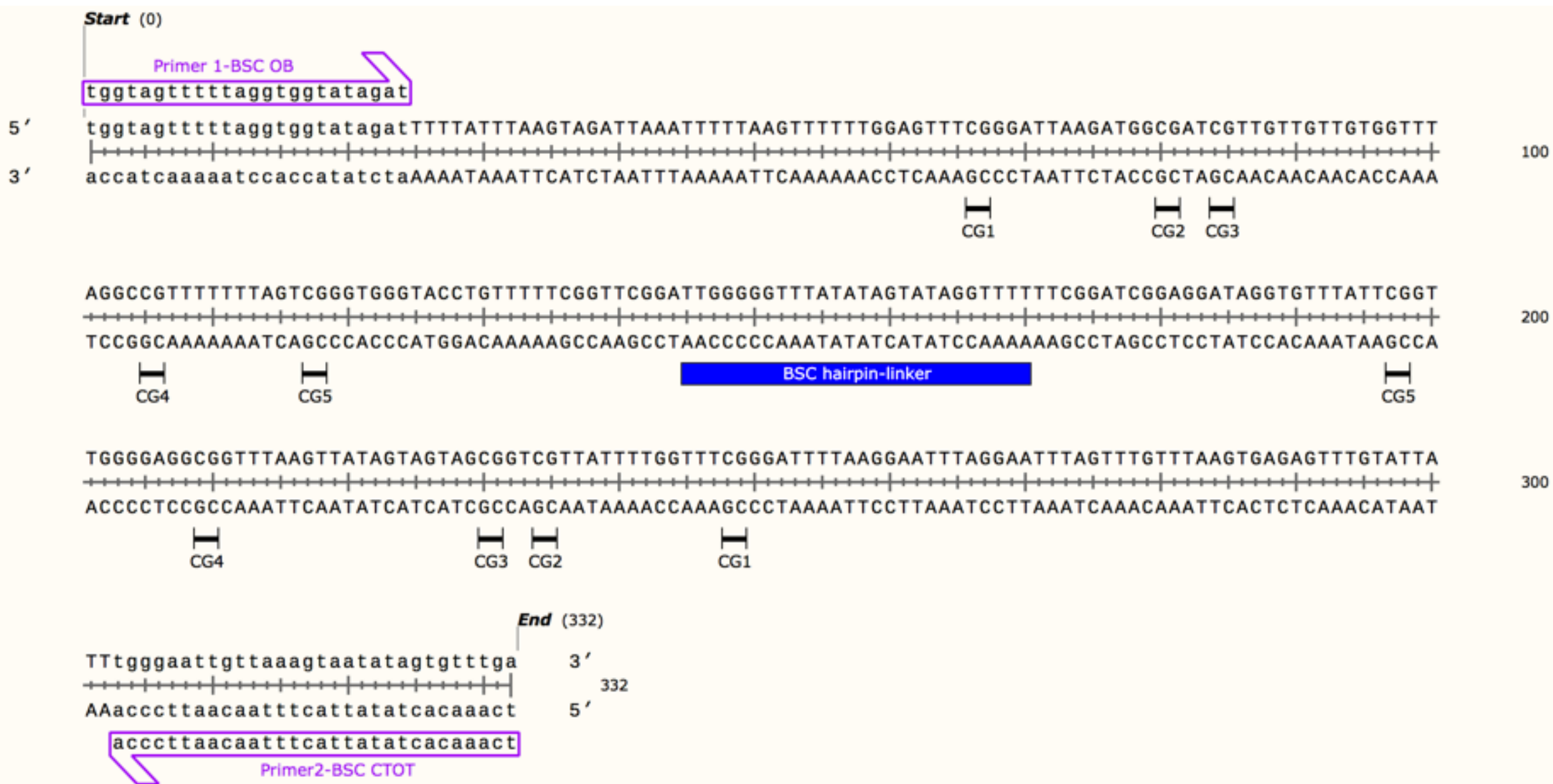

Supplement: Supplementary file 19 — Additional file 19: Figure S11. L1 hairpin-bisulfite sequencing amplicon analysis. Specific primers, Primer 1 and Primer 2 were used to amplify bisulfite-converted (BSC) and hairpin-linked L1MdTf promoter region. Primer 1 corresponds to BSC original top (OT) strand; Primer 2 corresponds to BSC complementary to original top (CTOT) strand. CpGs analyzed for hemimethylation with hairpin-bisulfite sequencing are indicated. [file 13072_2018_186_MOESM19_ESM.pdf]

**Fig. S12**

**A**

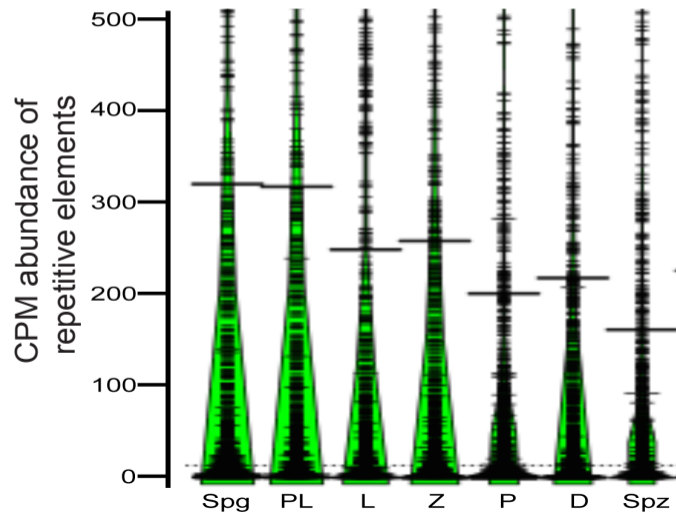

**B**

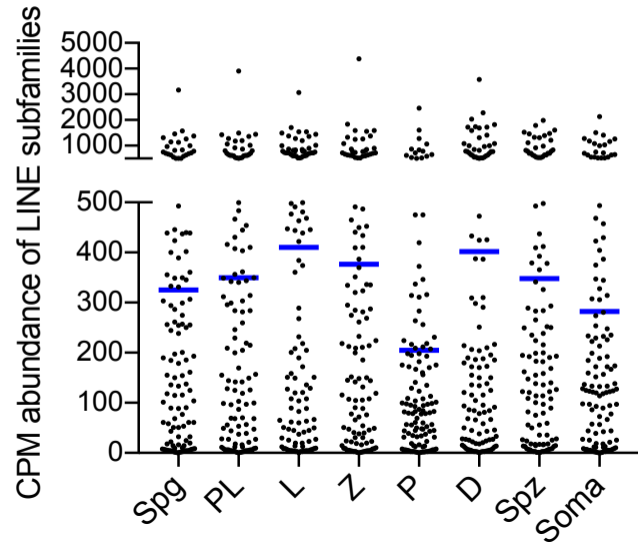

Supplement: Supplementary file 21 — Additional file 21: Figure S12. Analysis of transcript abundance of repetitive elements by RNA-seq. RepEnrich (fractional counts strategy) was used to calculate the total abundance of repetitive elements, expressed in counts per million mapping reads (CPM) for A) reads that map to all types of repeats annotated by repeat masker (n = 1266 types) and B) reads that map to LINE subfamilies (n = 121)(Supplemental Table S11). [file 13072_2018_186_MOESM21_ESM.pdf]

**Fig. S13**

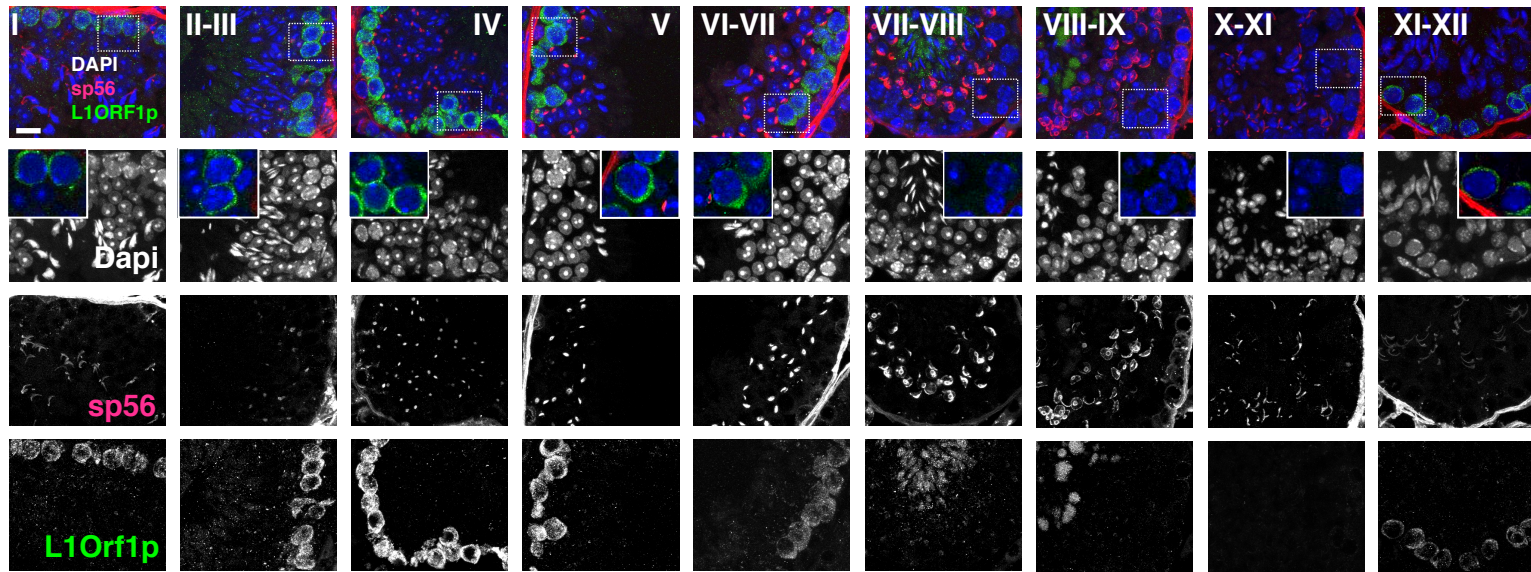

spermatid staging based on acrosome (sp56)

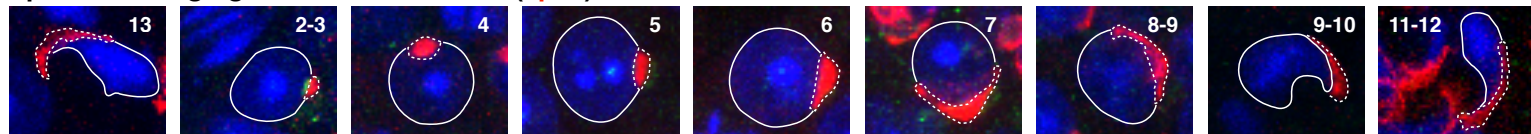

Supplement: Supplementary file 22 — Additional file 22: Figure S13. L1ORF1p expression in MPI of male germ cells. Temporal expression of L1ORF1p (green) was evaluated in testicular cryosections in the context of seminiferous epithelial cycle composed of stages I–XII. Haploid spermatids are identified based on numbers 1 through 16 according to degree of differentiation (only some are highlighted here). The basal membrane is outlined by the bright cross-reacting red staining. Following spermatogenic progression based on acrosome development marked by sp56 (red) and DNA stain, DAPI (blue), it is determined that cytoplasmic L1ORF1p is first seen in L/Z spermatocytes at stage XI (or X–XI) and persists from Z (stages XI–XII) to mid-P spermatocytes (stages I through VI–VII). L1ORF1p is not detectable in late P cells found stages VII–X, but is evident in the cytoplasm of elongating spermatids (see stages VII–IX) and is also detected as small dots in early round spermatids. The sp56 staining for spermatids beyond step 13 is difficult to see here, since the acrosome spreads very thin at this time. The selection inside the white box of the merged image (top row) is shown as a close-up inset in the DAPI-containing image row and represents a single confocal plane in an otherwise 3-D stacked image, highlighting the cytoplasmic distribution of L1ORF1p in meiotic prophase I spermatocytes. Bar = 10 micron. [file 13072_2018_186_MOESM22_ESM.pdf]
